# Cyclin A2 promotes DNA repair in the brain during both development and aging

**DOI:** 10.18632/aging.100990

**Published:** 2016-07-13

**Authors:** Patrick E. Gygli, Joshua C. Chang, Hamza N. Gokozan, Fay P. Catacutan, Theresa A. Schmidt, Behiye Kaya, Mustafa Goksel, Faisal S. Baig, Shannon Chen, Amelie Griveau, Wojciech Michowski, Michael Wong, Kamalakannan Palanichamy, Piotr Sicinski, Randy J. Nelson, Catherine Czeisler, José J. Otero

**Affiliations:** ^1^ Department of Pathology, The Ohio State University College of Medicine, Columbus, OH 43210, USA; ^2^ Mathematical Biosciences Institute, The Ohio State University, Columbus, OH 43210, USA; ^3^ Department of Neuroscience, The Ohio State University College of Medicine, Columbus, OH 43210, USA; ^4^ Department of Pediatrics, University of California, San Francisco School of Medicine, San Francisco, CA 94143, USA; ^5^ Department of Genetics, Harvard Medical School and Department of Cancer Biology, Dana Farber Cancer Institute, Boston, MA 02115, USA; ^6^ Department of Radiation Oncology, The Ohio State University College of Medicine. Columbus, OH 43210, USA

**Keywords:** Cyclin A2, DNA repair, mathematical modeling, neural stem cells, ventricular zone/subventricular zone

## Abstract

Various stem cell niches of the brain have differential requirements for Cyclin A2. Cyclin A2 loss results in marked cerebellar dysmorphia, whereas forebrain growth is retarded during early embryonic development yet achieves normal size at birth. To understand the differential requirements of distinct brain regions for Cyclin A2, we utilized neuroanatomical, transgenic mouse, and mathematical modeling techniques to generate testable hypotheses that provide insight into how Cyclin A2 loss results in compensatory forebrain growth during late embryonic development. Using unbiased measurements of the forebrain stem cell niche, we parameterized a mathematical model whereby logistic growth instructs progenitor cells as to the cell-types of their progeny. Our data was consistent with prior findings that progenitors proliferate along an auto-inhibitory growth curve. The growth retardation in *CCNA2*-null brains corresponded to cell cycle lengthening, imposing a developmental delay. We hypothesized that Cyclin A2 regulates DNA repair and that *CCNA2*-null progenitors thus experienced lengthened cell cycle. We demonstrate that *CCNA2*-null progenitors suffer abnormal DNA repair, and implicate Cyclin A2 in double-strand break repair. Cyclin A2's DNA repair functions are conserved among cell lines, neural progenitors, and hippocampal neurons. We further demonstrate that neuronal *CCNA2* ablation results in learning and memory deficits in aged mice.

## INTRODUCTION

The various compartments of the brain grow by distinct mechanisms. For example, the forebrain grows by expansion and radial migration of neural progenitor cells in the ventricular/subventricular zone (VZ/SVZ) that lines the lateral ventricles [1]. In contrast, the cerebellum is populated by cerebellar granule neuron progenitor cells of the external granule layer (EGL), which proliferate to expand the EGL, and then migrate inward to the internal granule layer [2]. We previously reported that loss of the S-phase Cyclin A2 (Gene symbol *CCNA2*; *Homo sapiens* accession number NM_001237.3; *Mus musculus* accession number NM_009828.2) results in cerebellar dysmorphia with relatively intact forebrain development [3]. This dichotomy raises an interesting question—why do the cells within these distinct stem cell niches respond differently to cell cycle dysfunction? Answers to similar questions have been proposed by non-traditional biological experiments. Specifically, mathematical modeling has been used to describe the dynamics of progenitor population size, using various methodologies [4-6]. Applied specifically to forebrain development, Takahashi et al. utilized measurements of cell cycle timing [7-9] to construct an empirical discrete-time model of the population size of the VZ/SVZ. This model was used to compute the thickness of the VZ/SVZ and surrounding regions from E11-E16. These seminal mathematical modeling studies demonstrated that the output of cell types from the cell niche varies during embryonic development, and proposed that only slight adjustments in cell fate change during embryonic development could change the quantity of neurons produced. Other groups have used this data to parameterize models of ordinary differential equations [10] and stochastic branching processes [11], although the large population size at E11 renders stochastic effects as negligible. These models however do not include a transient progenitor niche, as is known to exist [12], nor do they track the age of the cells or the transitions between phases of the cell cycle. They are also parameterized using biased measurements of VZ/SVZ thickness. Building upon this prior work, we sought to utilize mathematical modeling to help us understand how a *CCNA2-*null forebrain could develop grossly normal size and structure.

To assist in interpreting our neuroanatomical data, we developed a continuous-time mathematical model that incorporates aspects of this previous work as well as new data from our experiments presented in this study. Our model pays consideration to the transit of cells between cell cycle phases and the balance between proliferation and the production of intermediate progenitors. Indeed, many aspects of this model incorporate logistical growth concepts developed from the ecological sciences. Here, we used our model to explain how the forebrain VZ/SVZ stem cell population could overcome loss of Cyclin A2, whereas the cerebellar EGL does not. Our model is consistent with an overall mechanistic picture that *CCNA2* loss in the brain could be overcome through a developmental delay.

The components of the mathematical model include a lengthened cell cycle in *CCNA2*-null neural progenitor cells and a shift in the timing of production of intermediate progenitors and neurons. We treated the dynamics of the components of the model as testable hypotheses and confirmed that the cell cycle was indeed lengthened in *CCNA2*-null brains. We further confirmed that production of intermediate progenitors was decreased during early embryonic development and neuron production was increased post-natally, supporting our conclusion of a developmental delay.

One such cause of a lengthened cell cycle is unrepaired DNA damage, which induces cell cycle arrest. Our observation of an increased cell cycle time led us to ask if Cyclin A2 was involved in the DNA damage response. We found that Cyclin A2 is located at sites of DNA double-strand breaks (DSB) and plays a role in both homologous recombination repair and non-homologous end joining, the two major pathways for DSB repair.

These findings inspired us to investigate how *CCNA2* loss affected stem cell niches in adult animals. We did so by examining the effect of Cyclin A2 ablation in the adult hippocampus. We found that mice lacking Cyclin A2 had defects in DNA repair in embryonic progenitors and hippocampal neurons. Animals with the hippo-campal neuron pathologies showed concomitant reduction in performance in learning and memory tests. Taken together, our data underscores the importance of Cyclin A2 during both brain development and normal function of the adult brain and highlight the link between pathways common to both embryonic development and aging processes during adulthood. These data underscore the strength of mathematical modeling to elucidate new mechanistic insights to biological processes. Furthermore, our approach underscores the power of logistical growth modeling in the study of biological systems.

## RESULTS

### Cyclin A2 loss delays embryonic forebrain development

**Figure 1:**
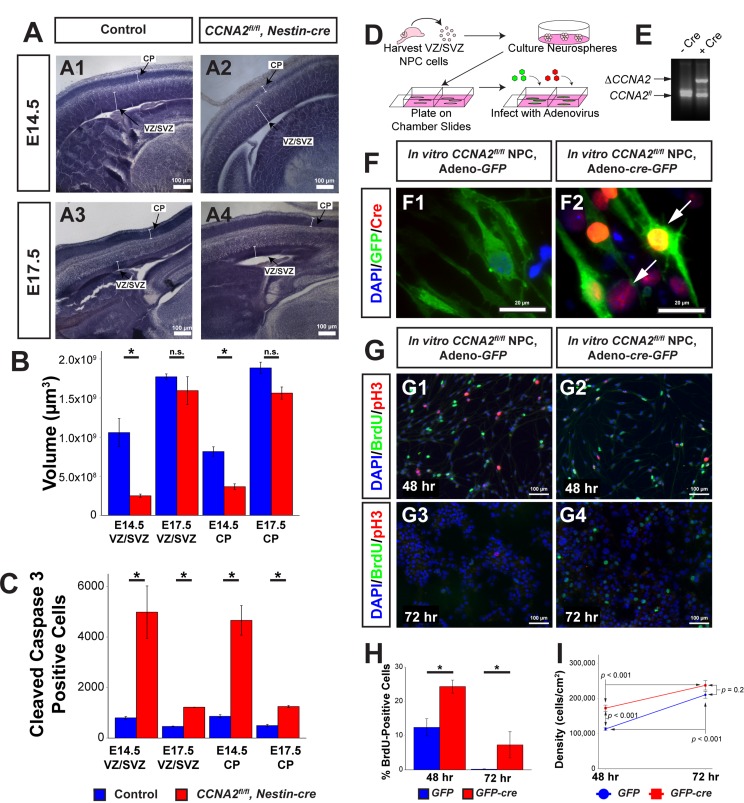
*CCNA2* Loss delays embryonic forebrain development (**A**) Representative low-magnification images used for unbiased stereology quantification. The VZ/SVZ and CP are noted by arrows. Experimental conditions are indicated above. (**B**) Total VZ/SVZ and CP volume. At E14.5, the volumes of both structures in *CCNA2^fl/fl^, Nestin-cre* animals were significantly reduced compared to controls. At E17.5, there was no statistical difference between groups. Quantifications represent Cavalieri unbiased stereology analysis of the entire brain. The *y*-axis is volume of the VZ/SVZ or CP. Unpaired *t*-test, * = *p* < 0.05, n.s. = not significant. For E14.5, n= 3 animals each for control and experimental groups. For E17.5, n=2 animals for control group and 3 animals for experimental group. (**C**) Total apoptotic cells in the VZ/SVZ and CP. At both ages, there was a significant increase in apoptosis in both structures. Quantifications represent Optical Fractionator unbiased stereology analysis of the entire brain at 100x magnification. The *y*-axis is total number of cleaved caspase 3-positive cells in the VZ/SVZ or CP. Unpaired *t*-test, * = *p* < 0.05. (**D**) Schematic of neural progenitor cell culture. Neural progenitors from the VZ/SVZ were dissected from P5 *CCNA2^fl/fl^* pups and cultured as neurospheres. Neurospheres were dissociated and infected with adenovirus encoding *cre* and *GFP* to excise *CCNA2*, or adenovirus encoding *GFP* only as a control. (**E**) *CCNA2^fl/fl^* ablation *in vitro*. DNA from infected cells was amplified by PCR. Alleles represented by each band are indicated on the left, and Cre condition is above. (**F**) Cells were stained for GFP and Cre recombinase. Infection of neural progenitor cells results in >90% infection. Arrows indicate Cre-positive cells in F2. (**G**) Cells were infected as shown in (**F**). Cells were pulsed with BrdU for 30 minutes before fixation 48 and 72 hours after plating. The proportion of BrdU and pH3-positive cells was increased in *CCNA2-*null cells. (**H**) Quantification of BrdU-positive cells 48 and 72 hours after plating. The *x*-axis is time after plating, and the *y*-axis is the percentage of cells that incorporated BrdU. Unpaired *t*-test, * = *p* < 0.05. (**I**) Quantification of cell density 48 and 72 hours after plating. Forty-eight hours after plating, *CCNA2*-null cells were less dense. There was no statistical difference between *CCNA2*-null and control cells 72 hours after plating. ANOVA with Tukey's HSD, *p* values are noted on the plot. These data support that *CCNA2-*null neural stem cells are capable of reaching the carrying capacity of their respective stem cell niches. The *x*-axis is time after plating, and the *y*-axis is cell density. Error bars for all graphs represent standard errors of the mean (s.e.m.).

In order to quantitatively describe the neuropathology of Cyclin A2 loss in the VZ/SVZ, we performed high-resolution analyses of the *CCNA2^−/−^* brains using unbiased stereological methodologies. We generated *CCNA2*-null brains by intercrossing *CCNA2^fl/fl^* mice with *Nestin-cre* mice. Ablation of *CCNA2* was confirmed by immunohistochemical staining (Fig. Supplemental S1). We focused our analyses on the VZ/SVZ of E14.5 and E17.5 mice. At E14.5, most radial glia divide symmetrically to expand the progenitor pool [13], while at later ages radial glia divide asymmetrically to self-renew and generate new neurons [14]. VZ/SVZ and cortical plate (CP) volumes and total number of cleaved caspase-3 positive cells in the entire VZ/SVZ and CP were determined in *CCNA2^fl/fl^*, *Nestin-cre* brains and compared to controls using unbiased stereology (Fig. 1A-C, Supplemental Table S1). E14.5 *CCNA2^fl/fl^*, *Nestin-cre* mice showed greater than 4-fold reduction in VZ/SVZ volume and greater than 2-fold reduction in CP volume (Fig. 1A-B). By E17.5, the CP and VZ/SVZ volumes were not significantly different between groups (*p* = 0.068 and *p* = 0.5, respectively). We conclude that during the E14.5->E17.5 period, the amount of growth of the *CCNA2^fl/fl^*, *Nestin-cre* VZ/SVZ was greater than that of the control VZ/SVZ.

To investigate the underlying cause of the early size reduction, we examined apoptosis in the VZ/SVZ and CP of these embryos by measuring the total number of cleaved caspase 3-positive cells in the VZ/SVZ and CP. We found greater than 5-fold increase in apoptosis in the VZ/SVZ and CP of *CCNA2^fl/fl^*, *Nestin-cre* embryos compared to controls at E14.5. E17.5 mice displayed approximately 2.5-fold increase in apoptosis in both structures (Fig. 1C). Notably, the volume occupied by apoptotic cells in the VZ/SVZ was vastly increased by *CCNA2* ablation compared to the increase observed in the cerebellar granule neuron progenitor cells of the external granule layer (Supplemental Table S1). These data indicate that forebrain neural progenitors are actually more sensitive to *CCNA2* ablation than cerebellar neuron progenitors, yet cerebellar morphogenesis is more adversely affected post-*CCNA2* ablation. To test if the observed growth delay was a property of neural progenitors in general, or was restricted to the embryonic period, we utilized an *in vitro* approach. We generated *CCNA2-*null neural progenitors dissected from the *CCNA2^fl/fl^* forebrain ganglionic eminence and expanded *in vitro*. *CCNA2* ablation was accomplished by infection with adenovirus encoding *cre-*recombinase. *CCNA2*-null neural progenitors showed increased BrdU incorporation, as well as delayed growth (Fig. 1F-I). Comparing the overall apoptosis rates to the overall proliferation rates, we concluded that the increase in apoptosis in the VZ/SVZ had minimal impact on the overall growth of the embryonic forebrain whereas apoptosis has more detrimental effects on cerebellar morphogenesis. Furthermore, we were perplexed by our finding of compensatory growth in *CCNA2*-null forebrains despite loss of a crucial cell cycle gene.

### Theoretical models of compensatory forebrain growth

We sought to understand the paradoxical effects of *CCNA2* ablation on embryonic neurogenesis. In particular, we interpreted the results from Fig. 1 in terms of the interplay between proliferation, self-renewal, apoptosis, and differentiation. The volume trajectories for both lines suggest autoregulatory saturation of the neuronal population by day E15. This plateau is similar to that shown in previous observations in the literature [8, 15]. The simplest model of such population dynamics is logistic growth*^1^, where proliferation rate decreases as a population approaches a limiting capacity. Logistic growth has been shown to well-explain the growth of many tissues including in the brain [16]. Since the main feature of logistic growth is a decrease in proliferation rate as a population approaches a certain threshold or carrying capacity*, two populations that initially grow at the same rate may converge to the same steady size over a sufficient amount of time. For this reason, it provides the most simplistic mechanism for the slow-down in the normal growth rate that would allow the *CCNA2*-null embryos to catch up to their *CCNA2*-intact counterparts.

**Figure 2:**
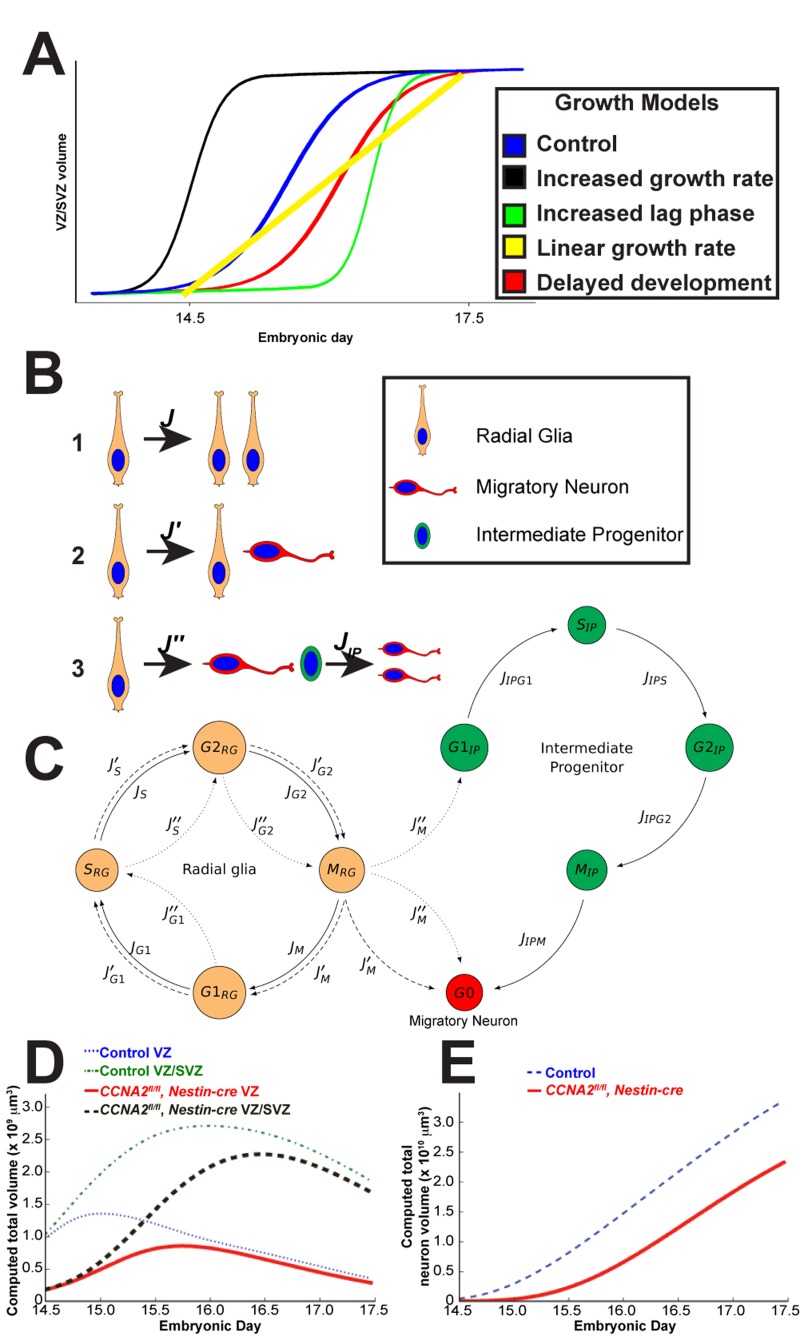
Mathematical modeling of forebrain growth (**A**) Hypothetical models of forebrain growth. Various possible explanations of forebrain growth in *CCNA2^fl/fl^, Nestin-cre* brains are presented. (**B**) Schematic of the fates of a radial glia neural stem cell in our model. Radial glia can divide in (1) a symmetric self-renewal, (2) an asymmetric self-renewal generating a migratory neuron and a radial glia, or (3) an asymmetric, non-renewing division generating a migratory neuron and an intermediate progenitor, with the intermediate progenitor dividing into two migratory neurons. (**C**) Diagram of cell cycle progression corresponding to our mathematical model. Cells leave each phase of the cell cycle at different rates *J*_(·)_ depending on population size and mitotic age. These parameters are described in Supplemental Table S4. Cells are also able to enter an apoptotic state from each phase. The flows between the cell cycle phases for each type of division in (**A**) are noted by *J*, *J'*, *J''*, or *J_IP_*. (**D**) Computed volumes of the VZ and combined VZ/SVZ for *CCNA2^fl/fl^, Nestin-cre* and control simulations. The control VZ volume plateaus at approximately E15, whereas it continues to grow in the *CCNA2^fl/fl^, Nestin-cre* until E15.5. Similar trends are seen in the combined VZ/SVZ volumes. The *x*-axis is the embryonic day, and the *y*-axis is the volume of the VZ or combined VZ/SVZ. (**E**) The cumulative neuronal output of the VZ/SVZ during E14.5-E17.5 of the *CCNA2^fl/fl^, Nestin-cre* simulations trails that of the control throughout the time period. The *x*-axis is embryonic day, and the *y*-axis is total neuronal volume.

In order to better understand the growth of *CCNA2^fl/fl^*, *Nestin-cre* brains, we considered several hypotheses to explain the data. These hypotheses are presented as theoretical growth curves in Fig. 2A. The blue curve represents logistic growth of *CCNA2*-intact brains. Growth mediated by more rapid cell cycle (black curve) cannot explain the growth of *CCNA2^fl/fl^*, *Nestin-cre* brains as it would require the *CCNA2^fl/fl^*, *Nestin-cre* VZ/SVZ volume to be increased at E14.5, in addition to showing more rapid cell cycle transit in cells lacking a critical S-phase cyclin. Elevated apoptosis would result in a prolonged lag phase (green curve), yet our measurements of apoptosis do not show a sufficiently elevated programmed cell death rate to cause such a lag. Thus, an increased lag phase model cannot explain the growth of *CCNA2^fl/fl^*, *Nestin-cre* brains because it would require the apoptosis rates to be much higher than what we observed in order to delay the transition from lag to log phase. The linear growth model (yellow curve), corresponding to a fixed-size proliferative pool, cannot explain the growth of *CCNA2^fl/fl^*, *Nestin-cre* brains because it would either require apoptosis rates to be high relative to proliferation rates, which is not the case, or it would require that the VZ/SVZ stem cell niche be populated only by a single cell type [1].

Our considerations of these alternative models left us with only our delayed development model (red curve) to explain the data. To test this hypothesis, we generated a mathematical model of mouse forebrain development using previously reported data on cell cycle timings available in the scientific literature, as well as data from our own unbiased estimates of VZ/SVZ size during embryonic development (Fig. 1 and 2, and Supplemental Tables S2-S4). The variables that we track in our model are the total volumes of cells in each of the G1/S/G2/M phases, existing within each of the VZ/SVZ niches, which have a given mitotic age. The parameters in this model are the rates of transitions between each stage of the cell cycle. We incorporated logistic growth mechanism in determining cell fate, with the rate of Type I pro-liferative divisions (Fig. 2B) decreasing linearly as a function of total volume.

Assuming a conservatively rapid clearance rate for apoptotic cells, we found that apoptosis did not significantly affect the population dynamics* in the E14.5 to E17.5 period. Although the apoptosis rate increases many-fold, the apoptosis rate remains mathematically insignificant relative to the growth rate (Supplemental Table S1). Since we have determined that apoptosis is insignificant, a decrease in growth rate is likely the determinant of the observed developmental delay. For this reason, we expected that *CCNA2* loss in a neural stem cell lengthens the cell cycle, a finding that has previously been identified in *CCNA2*-null mouse embryonic fibroblasts [17]. We assume that such cell cycle lengthening occurs during S and G2 phases as these are the phases during which Cyclin A2 protein is present.

Simulations of the VZ/SVZ volume are given in Fig. 2D, where we assumed that the SVZ has no volume at E13.5, and that all radial glia present at E13.5 have the capability to divide (on average) approximately five more times before terminally differentiating. We arrived at the number five in order to match the characteristics of the observations in our unbiased measurements for the *CCNA2*-intact controls. We then tested simulations as to how a delayed forebrain stem cell niche would behave.

At E14.5, the measured control VZ/SVZ volume is approximately 4 times that of *CCNA2^fl/fl^*, *Nestin-cre.* Supposing that this *cre* expresses at E10.5 [18], and that the divisions in this period are primarily Type I symmetric divisions (Fig. 2B), which have a mean transit time of 9.2 hrs, then proliferative *CCNA2^fl/fl^*, *Nestin-cre* radial glia have a mean transit time of 10.6 hrs. Isolating the excess time of 1.4 hrs to the S and G2 phases suggests that they are elongated by 25%. This also suggests that at E13.5, *CCNA2^fl/fl^ Nestin-cre* radial glia are capable on average of approximately 1 more division than in control animals. For this reason, we assumed that the radial glia are developmentally delayed, giving them the capability to divide six times before terminal differentiation during the E14.5-E17.5 time period. These simulations thus shed light as to why increased forebrain apoptosis does not affect overall growth rate in the *CCNA2^fl/fl^*, *Nestin-cre* animals.

In summary, the notion that developmental delay results in compensatory growth in *CCNA2^fl/fl^ Nestin-cre* is best explained by a predominance of symmetric divisions (Type 1 in Fig. 2B) that expand the VZ/SVZ between E14.5 and E17.5, while neural progenitors in the control brains undergo more asymmetric and terminal divisions, slowing the growth of the control VZ/SVZ. Stated otherwise, the *CCNA2^fl/fl^ Nestin-cre* brains behave similarly to control brains, although delayed by approximately one day.

**Figure 3:**
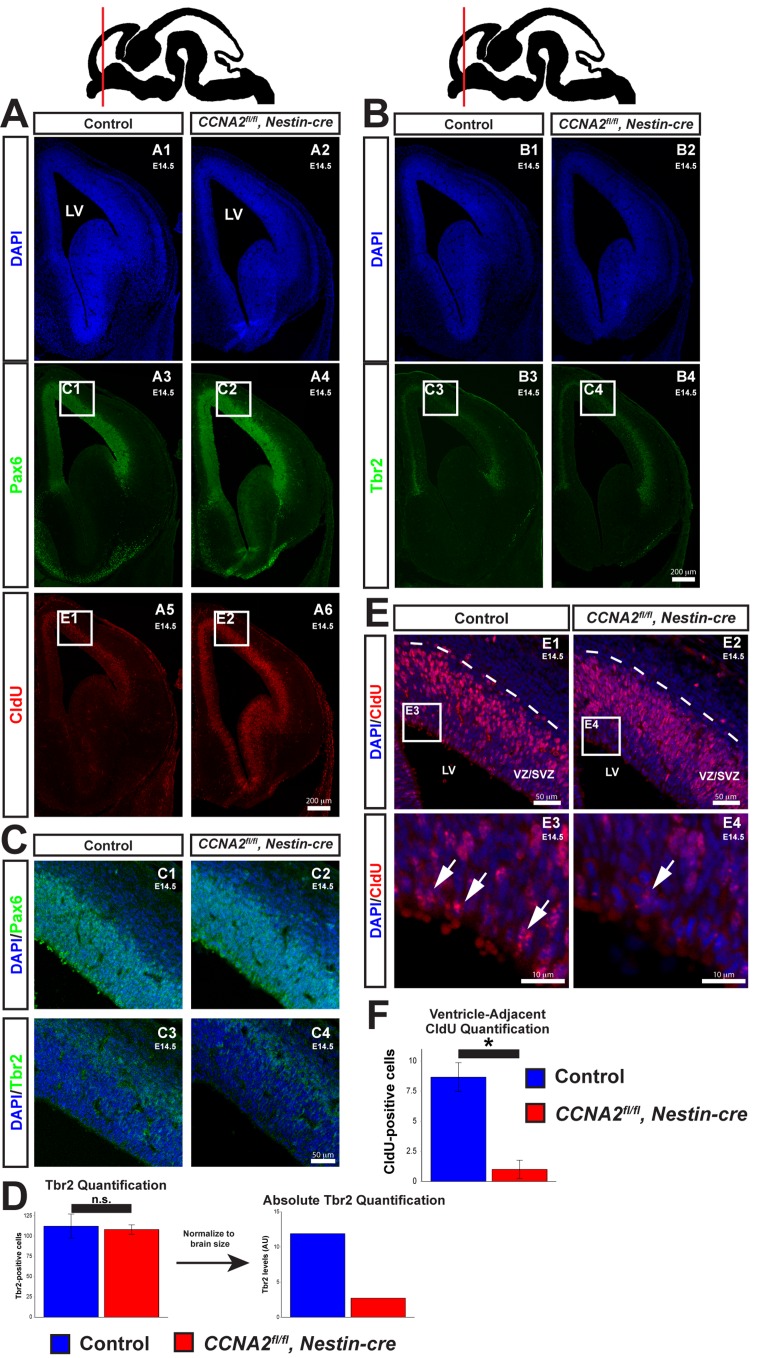
Delayed forebrain development in *CCNA2^fl/fl^*, Nestin-cre animals (**A**) A timed-pregnant E14.5 dam was injected with CldU then euthanized. Embryos were stained for Pax6, CldU, and DAPI. Experimental condition is indicated above, molecular markers are color-coded on the left. LV = lateral ventricle. (**B**) Sections were stained for Tbr2. Experimental condition is indicated above, molecular markers are color-coded on the left. (**C**) Pax6 and Tbr2-positive cells are appropriately localized in *CCNA2^fl/fl^, Nestin-cre* forebrains, indicating preserved cytoarchitecture. Image locations are indicated in (**A**) and (**B**). (**D**) Quantification of Tbr2-positive cells. Cells were counted in a 100×180 μm counting frame in the VZ/SVZ. Tbr2-positive cells were unchanged within each counting frame. Accounting for the reduced size of the E14.5 VZ/SVZ there is a reduction in total Tbr2-positive cells. The *y*-axis is total Tbr2-positive cells. Unpaired *t*-test, n.s. = not significant, *p* > 0.05, n = 3 embryos per condition. (**E**) High magnification images of CldU staining in (**A**). Experimental condition is indicated above, molecular markers are color-coded on the left. Arrows indicate CldU-positive cells adjacent to the ventricle. (**F**) Quantification of CldU-positive cells adjacent to the lateral ventricle. CldU-positive cells were counted in a 100×10 μm bin adjacent to the ventricle. CldU-positive cells adjacent to the lateral ventricle were reduced in *CCNA2^fl/fl^, Nestin-cre* embryos. The *y*-axis is number of CldU-positive cells. Unpaired *t*-test, *p* < 0.05, n = 3 embryos per condition. Error bars for all graphs represent s.e.m.

Our developmental delay model's predictions include (1) that the *CCNA2-*null neural progenitor cells have a prolonged cell cycle time, (2) the total output of intermediate progenitors would be decreased early in development, (3) more neurons would be produced at later time points, and (4) at later time points the mutants would have a higher overall level of proliferation. In order to test the predictions of our model, we examined VZ/SVZ cytoarchitecture and proliferation in E14.5 embryos and P0 pups. We injected an E14.5 timed-pregnant mouse with CldU, euthanized the pregnant dam 2.5 hours later, and harvested embryos. We first examined VZ/SVZ cytoarchitecture in these embryos. Neural progenitor cells are characterized by expression of Pax6, localized adjacent to the ventricle. As they divide and mature into intermediate progenitors, they express Tbr2 and are located deeper within the VZ/SVZ [19]. We observed appropriately-localized Pax6 and Tbr2, indicating that loss of *CCNA2* does not perturb VZ/SVZ cytoarchitecture (Fig. 3A-C). We quantified Tbr2 and found that there was no change in the number of Tbr2-positive cells in individual sections (Fig. 3D). However, in concordance with our model, there was a reduction in the total number of Tbr2 cells when we normalized the volumes for the smaller size of the *CCNA2^fl/fl^*, *Nestin-cre* VZ/SVZ (Fig. 3D).

To confirm that the cell cycle was lengthened in *CCNA2^fl/fl^, Nestin-cre* neural progenitors, we examined the localization of CldU-positive nuclei within the VZ/SVZ. Neural progenitors undergo interkinetic nuclear migration (INM), a process in which the localization of the nucleus within the cell depends upon cell cycle phase. During S-phase, the nucleus is located away from the ventricle, deep within the VZ/SVZ (Fig. 3A, E). Upon completion of DNA synthesis, the nucleus migrates back to the ventricular surface, where the cell divides. Cells located outside the prominent band of CldU-positive nuclei deep in the SVZ would therefore have completed S-phase and begun INM toward the ventricle. Increasing the duration of S phase would result in fewer CldU-positive nuclei that have completed DNA synthesis and begun their migration back to the ventricular surface. With this in mind, we measured the number of CldU-positive nuclei adjacent to the ventricle. Very few of these cells are noted in *CCNA2^fl/fl^, Nestin-cre* embryos compared to controls (Fig. 3E-F), indicative of a lengthened S-phase. In summary, *CCNA2* loss results in increased brain growth during E14.5->E17.5, despite showing prolonged cell cycle time and increased apoptosis.

Between E14.5 and E17.5, wild-type VZ is known to plateau and decay due to differentiation [8, 15]. Due to this normal growth plateau, the *CCNA2^fl/fl^*, *Nestin-cre* VZ recovers in volume relative to the control. This recovery, however, is misleading, as the cumulative neuronal output is predicted to be significantly depressed (Fig. 2E) in the mutant animals. Indeed, our model suggests that the CNS stem cell niche would exhibit behaviors at P0 characteristic of earlier embryonic stages. Stated otherwise, proliferation in *CCNA2^fl/fl^*, *Nestin-cre* animals would remain elevated and a significant number of neurons would be generated later in development from the forebrain stem cell niche.

**Figure 4:**
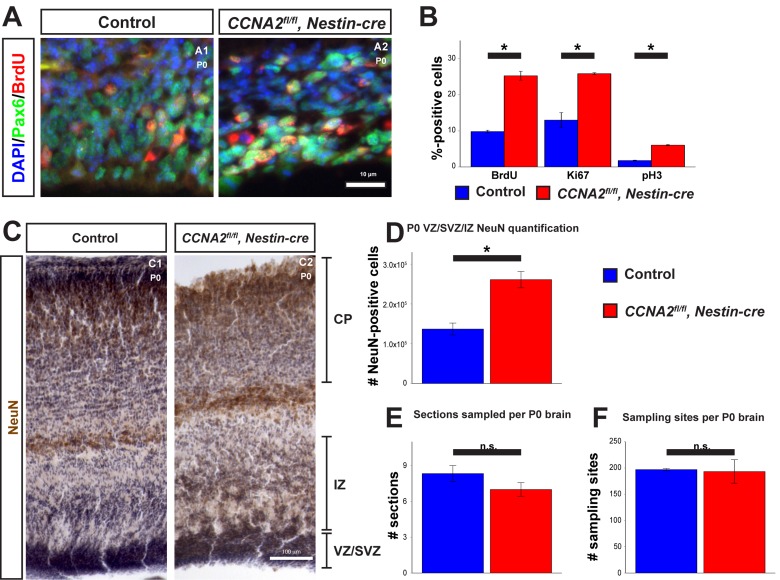
Newborn *CCNA2^fl/fl^*, Nestin-cre animals exhibit a developmental delay (**A**) P0 animals were pulsed with BrdU for 30 minutes before euthanasia and tissue stained for BrdU and Pax6. (**B**) Quantifications of BrdU, Ki67, and pH3 were significantly elevated in *CCNA2^fl/fl^, Nestin-cre* mice. The *y*-axis is the percentage of cells positive for each marker in the VZ/SVZ. Unpaired *t*-test, * = *p* < 0.05. (**C**) P0 brains were sectioned for unbiased stereology, stained for NeuN, and counterstained with hematoxylin. NeuN-positive cells were quantified in the combined VZ/SVZ and intermediate zone (IZ). Experimental conditions are indicated above, molecular marker is color-coded on the left. VZ/SVZ, intermediate zone (IZ), and CP are indicated. (**D**) Optical fractionator measurements of NeuN-positive cells in the VZ/SVZ/IZ show increased neuronal output in *CCNA2^fl/fl^, Nestin-cre* animals compared to controls, indicative of a developmental delay. The *y*-axis is the number of NeuN-positive cells in the VZ/SVZ/IZ per brain. Unpaired *t*-test, * = *p* < 0.05, n = 3 brains per condition. (**E**) Measurement of the number of sections sampled and (**F**) measurement of the number of sampling sites. There was no significant difference in either metric, precluding the possibility that the increased NeuN was due to a difference in brain size. For (**E**), the *y*-axis is the number of sections sampled per brain. For (**F**), the *y*-axis is the number of sampling sites per brain. Unpaired *t*-test, n.s. = not significant, *p* > 0.05, n = 3 brains per condition. Error bars for all graphs represent s.e.m.

To test these predictions, we examined proliferation in the VZ/SVZ of P0 mice. We injected P0 mice with BrdU and euthanized them 30 minutes later. We then stained for BrdU (Fig. 4A), pH3, and Ki67. Consistent with our model, we observed an increase in all three of these proliferation markers in the VZ/SVZ of *CCNA2^fl/fl^*, *Nestin-cre* mice (Fig. 4B). Furthermore, we measured neuronal output in newborn mice by determining the total number of NeuN-positive cells in the combined VZ/SVZ and intermediate zone (IZ) with unbiased stereological quantification of NeuN immunohistochemistry. We found that the total number of neurons in the combined VZ/SVZ and intermediate zone (IZ) was significantly increased, as predicted by our model (Fig. 4C-F). Thus, we conclude that *CCNA2* loss does not affect stem cell self-renewal or migration to the CP and the forebrain develops appropriately, yet remains developmentally immature relative to a control brain. Furthermore, our experimental data and our mathematical model predict a maximal limit to the size of the forebrain stem cell niche, which we term the carrying capacity (*V_max_*). *V_max_* is constant between the control and *CCNA2^fl/fl^*, *Nestin-cre* brains. Since at E14.5 the *CCNA2*-null neural progenitor cells are at a different point in the growth curve toward *V_max_*, our model predicts that levels of proliferative cells would be elevated relative to controls as the *CCNA2*-null cells would not experience the autoinhibitory mechanisms acting on the control stem cell niche during equivalent epochs.

### Mechanisms of increased cell cycle time in CCNA2 deficient cells

**Figure 5:**
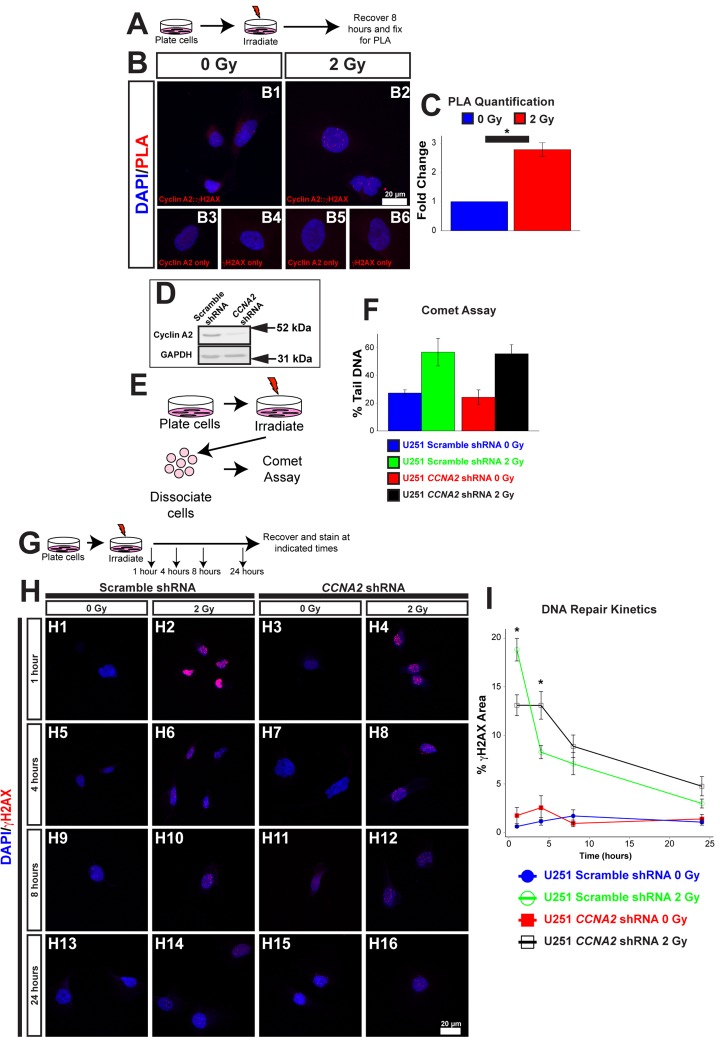
γH2AX is located at foci of DNA damage (**A**) Schematic of experiment in (**B**) and (**C**). U251 cells were irradiated with 2 Gy. 8 hours after irradiation, cells were processed for PLA. (**B**) Association between Cyclin A2 and γH2AX. Positive PLA signals manifest as punctate foci in the nucleus. Single-labeled controls are presented below. Experimental conditions are indicated above, molecular markers are color-coded on the left. Antibodies used are indicated in each image. (**C**) Quantification of (**B**). The *y*-axis is fold-change of PLA signals induced by radiation. 2 Gy induces associations between γH2AX and Cyclin A2 approximately 3-fold. Unpaired *t*-test, * = *p* < 0.05, n=3 independent experiments, 45 total cells for 0 Gy condition, 44 total cells for 2 Gy condition. (**D**) Silencing of *CCNA2* by shRNA confirmed by western blotting. (**E**) Schematic of comet assay. (**F**) Equivalent DNA damage after treatment with X-rays in *CCNA2-*silenced U251 cells. Comet assay was performed on cells with *CCNA2* or scramble shRNA after treatment with 2 Gy X-rays. We did not observe a significant change in DNA damage (ANOVA, n=2). The *y*-axis shows percentage of DNA found in the tail of each comet. (**G**) Schematic of experiment in (**H**) and (**I**). U251 cells encoding either *CCNA2*-targeting or scrambled shRNA were irradiated with 2 Gy or mock-treated. Cells were allowed to recover and fixed the times indicated then stained for Rad51 and γH2AX. (**H**) Silencing *CCNA2* reduces phosphorylation of H2AX after 2 Gy and slows DNA repair rates as measured by γH2AX signal as a percentage of the nuclear area. (**I**) *CCNA2*-silenced cells display decreased phosphorylation of H2AX and delayed DNA repair. The *x*-axis is the time after irradiation, and the *y*-axis is the area of each nucleus with γH2AX signal. ANOVA with Tukey's HSD, * = *p* < 0.05 between the *CCNA2* and scramble shRNA, n=3 independent experiments, 46-55 total cells per condition. Error bars for all graphs represent s.e.m.

The lengthening of the cell cycle in *CCNA2^fl/fl^*, *Nestin-cre* mice is perhaps not surprising, given the loss of an important cell cycle regulator. However, Cyclin E has been shown previously to compensate for loss of Cyclin A2 [17], leading us to ask if there were other underlying reasons for the lengthened cell cycle. One of the major causes of cell cycle arrest leading to a lengthened cell cycle is DNA damage. The function of Cyclin A2 with its effector kinase CDK2 is blocked during the DNA damage checkpoint, which stops progression through the cell cycle [20]. However, our results suggest that Cyclin A2 may have a more active role in the DNA damage response. Therefore, we asked if Cyclin A2 was involved in promoting DNA repair in addition to its well-characterized cell cycle roles. To answer these biochemical questions, we utilized an *in vitro* cell culture model. First, we tested the extent to which Cyclin A2 localized to sites of DNA damage after ionizing radiation (IR), which induces DNA double-strand breaks (DSBs). Cells were exposed to 2 Gy of X-ray radiation, and the localization of Cyclin A2 with the DNA double-strand break marker γH2AX was tested by proximity ligation assay (PLA). Epitopes located within 30 nm of each other elicit fluorescent punctae [21], which we quantified by automated counting in ImageJ. We observed an approximately three-fold increase in PLA signals in the nuclei during recovery from X-irradiation (Fig. 5A-C), which is indicative of localization of Cyclin A2 at double-strand breaks. We conclude that Cyclin A2 is found at DSB sites after ionizing radiation.

We next tested the hypothesis that *CCNA2* played a role in the signal transduction cascade of the DNA damage response. One of the main hallmarks of the DNA signal transduction cascade is phosphorylation of H2AX [22]. In normal cells, IR treatment results in acutely high levels of phosphorylated H2AX (γH2AX); the levels of this phosphorylated histone returns to baseline following DNA repair. We therefore asked if the phosphorylation of H2AX was affected by loss of Cyclin A2. We used a previously published and validated lentiviral approach to silence *CCNA2* expression (Fig. 5D) [23]. Comet assay found no change in DNA damage between *CCNA2*-silenced cells and control cells (Fig. 5E-F). We then analyzed levels of γH2AX in *CCNA2*-silenced cells. Quantification of γH2AX normally involves counting of nuclear foci. In our case, we observed such a large γH2AX response to irradiation that we were unable to count distinct foci; we thus analyzed the percent of the nucleus that was positive for γH2AX signal. *CCNA2*-silenced cells phosphorylated H2AX post-IR at lower levels than did control cells. Furthermore, *CCNA2-*silenced cells showed delayed DNA repair kinetics 1 and 4 hours post-IR, indicating that the damaged DNA was repaired at a reduced rate (Fig. 5G-I). Thus, reduced Cyclin A2 protein levels results in decreased H2AX phosphorylation with a functional, albeit slowed, DNA damage response.

**Figure 6:**
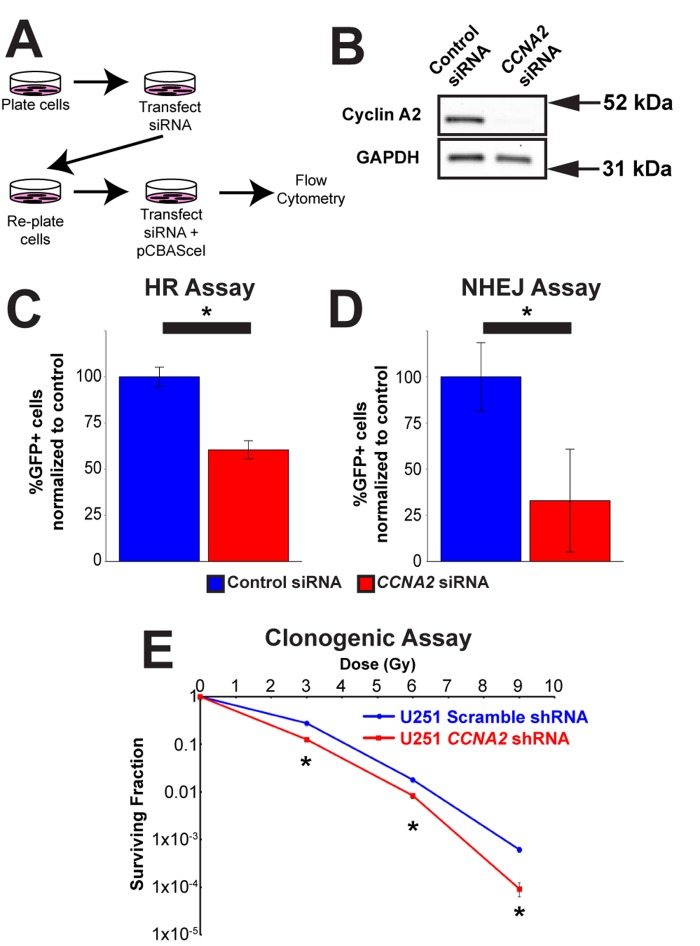
Cyclin A2 is involved in both homologous recombination and non-homologous end joining (**A**) Schematic of experiments in (**C**) and (**D**). (**B**) Silencing of *CCNA2* by siRNA was confirmed by western blot. (**C**) siRNA silencing of *CCNA2* reduces HR. U251 cells with an integrated pDR-GFP plasmid were transfected with pCBASceI to induce a DSB in the DR-GFP locus. Cells that repaired this DSB by HR express GFP. Percentage *CCNA2*-silenced cells expressing GFP were normalized to percentage of control cells expressing GFP. Unpaired *t*-test, * = *p* < 0.05, n=3 independent experiments. (D) Silencing of *CCNA2* reduces NHEJ. Experiments were performed as in (**C**), but with an integrated EJ5GFP plasmid. Unpaired *t*-test, * = *p* < 0.05, n=4 independent experiments. The *y*-axes in (**C**) and (**D**) are the percentage of GFP-positive cells in each condition normalized to the control siRNA condition transfected with pCBASceI plasmid. Error bars represent SEM for all graphs. (**E**) Lentiviral silencing *CCNA2* sensitizes cells to IR by clonogenic assay. *CCNA2*-silenced cells demonstrate reduced survival compared to control cells with scrambled shRNA. The *x*-axis is dose, and the *y*-axis is surviving fraction. Unpaired *t*-test at each dose, * = *p* < 0.05, n=3 independent experiments. Error bars for all graphs represent s.e.m.

With this in mind, we tested the hypothesis that *CCNA2* was a mediator of DSB repair by performing DNA repair assays in *CCNA2-*silenced cells. Specifically, we utilized the DRGFP homologous recombination (HR) and EJ5GFP non-homologous end joining (NHEJ) assays [24, 25], which required us to modify our *CCNA2-*silencing methodology (Fig. 6A-B). In these assays, DSB resolution by the pathway in question is determined by manifestation of GFP fluorescence detected by flow cytometry. *CCNA2*-silenced cells showed a mean reduction of 40% in HR and a mean reduction of 67% in NHEJ (Fig. 6C-D and Fig. Supplemental S2). These data are in line with reductions in GFP fluorescence observed by silencing other DNA repair genes such as RNF8 [26]. *In toto,* our finding showing blunted γH2AX formation and deficient HR and NHEJ DSB repair in *CCNA2-*silenced cells support the notion that *CCNA2* functions early in the DSB signal transduction cascade. Such a finding would result in radiation sensitivity in *CCNA2-*silenced cells. Lentiviral *CCNA2-*silenced cells were thus subjected to the clonogenic assay [27] after exposure to 0, 3, 6, or 9 Gy of X-rays. Cells deficient in *CCNA2* were significantly more sensitive than control cells (Fig. 6E). We conclude that DSB repair is deficient in *CCNA2* silenced cells.

### *CCNA2*-null neural progenitor cells of the VZ/SVZ show evidence of intrinsic DNA repair defects

**Figure 7:**
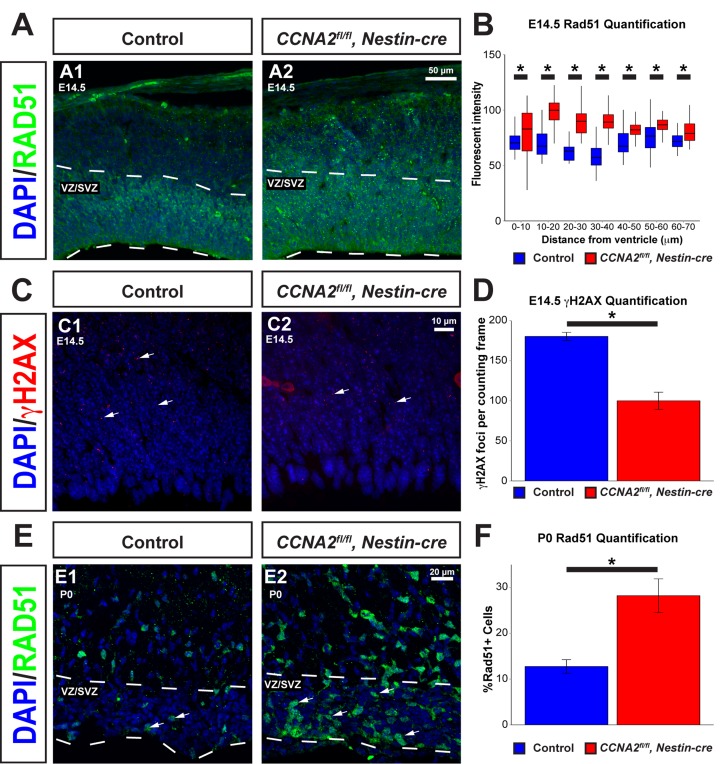
Rad51 levels are increased in the VZ/SVZ of *CCNA2^fl/fl^*, Nestin-cre animals (**A**) Cryosections from E14.5 brains were stained for Rad51. The VZ/SVZ is denoted by a dashed line. (**B**) Quantification of Rad51 levels in the E14.5 VZ/SVZ shown in (**A**). Profile plots of fluorescence intensity in 10 μm bins from the ventricle show increased Rad51 levels in the VZ/SVZ. The *y*-axis is Rad51 fluorescence intensity, and the *x*-axis is distance from the ventricle. Unpaired *t*-test, * = *p* < 0.05, n=3 animals per condition. (**C**) Sections of E14.5 brains were stained for γH2AX. Arrows indicate foci of γH2AX. (**D**) Quantification of γH2AX levels in the E14.5 VZ/SVZ shown in (**C**). γH2AX foci are decreased in *CCNA2^fl/fl^, Nestin-cre* animals compared to controls. γH2AX foci were counted in a standard 50×90 μm counting frame. The *y*-axis is γH2AX foci per counting frame. Unpaired *t*-test, * = *p* < 0.05, n=3 animals per condition. (**E**) Cryosections of P0 brains were stained for Rad51. Arrows indicate Rad51-positive cells. The VZ/SVZ is denoted by a dashed line. (**F**) Quantifications of images from (**E**). Percentage of cells expressing Rad51 were counted and normalized to total nuclei in the VZ/SVZ. The percentage of Rad51-positive cells is increased in *CCNA2^fl/fl^, Nestin-cre* brains. The *y*-axis is the percentage of cells in the VZ/SVZ that express *RAD51*. Unpaired *t*-test, * = *p* < 0.05, n=2 animals per condition. Error bars for all graphs represent s.e.m.

We next asked if loss of Cyclin A2 had a similar effect in neural progenitors found in the VZ/SVZ forebrain stem cell niche. To achieve this, we evaluated E14.5 embryos and P0 pups in which *CCNA2* was ablated in neural progenitors by intercrossing *CCNA2^fl/fl^* mice with *Nestin-cre* mice. We examined the VZ/SVZ of these animals for hallmarks of DNA damage experienced during normal development. Such DNA damage causes cell cycle arrest, resulting in a slowed cell cycle. A common physiological cause of cell cycle arrest includes DSB formation caused by replication fork collapse or generation of reactive oxygen species (reviewed in [28, 29]). With this in mind, we examined VZ/SVZ neural progenitor cells *in vivo* for increased Rad51, a homologous recombination protein elevated in cells undergoing HR [30]. We noted an increased proportion of VZ/SVZ neural progenitor cells expressed *RAD51* in *CCNA2^fl/fl^*, *Nestin-cre* animals at both E14.5 and P0, supporting the notion that these cells were undergoing DNA repair (Fig. 7A-B, E-F). This also supports the notion that loss of Cyclin A2 does not fully block DNA repair, but decreases the rate of repair as evidenced by the persistence of Rad51 expression in cells outside the VZ/SVZ. We then investigated if *CCNA2^fl/fl^*, *Nestin-cre* neural progenitor cells showed altered DNA damage (Fig. 7C-D). We quantified γH2AX in the dorsal VZ/SVZ and observed a reduction in the mean number of γH2AX foci, in concordance with our cell culture data (Fig. 5J-K). This is in contrast to brains deficient in NBS and ATM, two high-level regulators of the DNA damage response that showed an increase in γH2AX in the VZ/SVZ during embryonic development [31]. This further supports the importance of H2AX phosphory-lation on Cyclin A2 function. We conclude that *CCNA2*-null neural progenitor cells show evidence of active DSB repair *in vivo*.

### *CCNA2*-null cells of the hippocampus show evidence of intrinsic DNA repair defects

**Figure 8:**
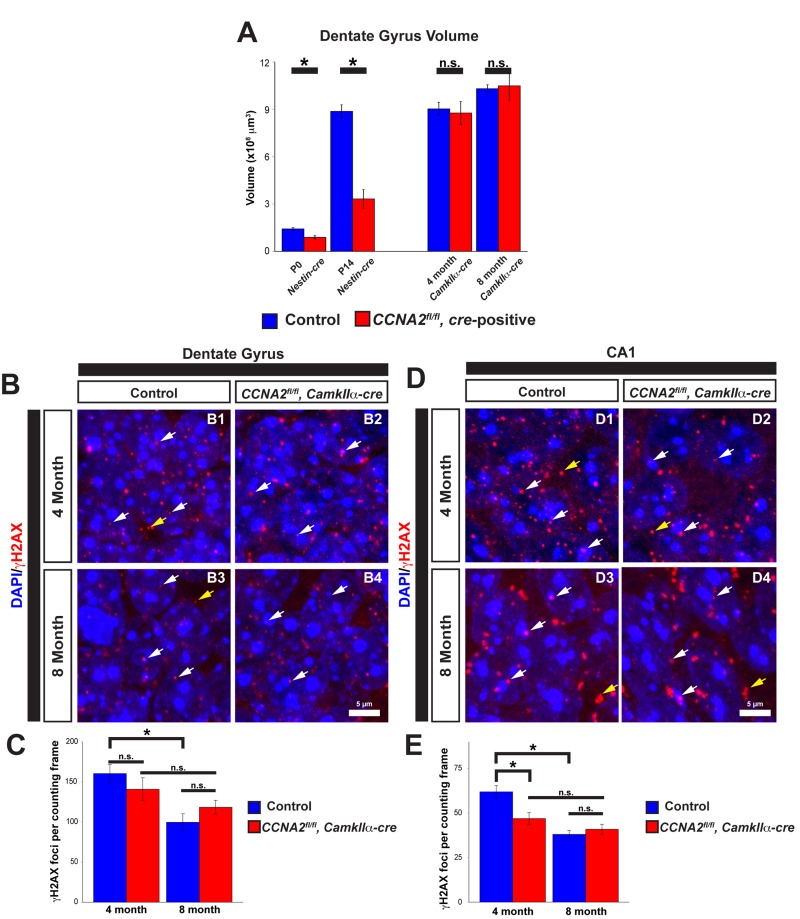
Cyclin A2 loss impairs hippocampal development (**A**) Cavalieri estimations of the size of the dentate gyrus were performed in *CCNA2^fl/fl^, Nestin-cre* brains at P0 or P14, and in *CCNA2^fl/fl^, CamkIIa-cre* brains at 4 months or 8 months of age. The *y*-axis is volume of the dentate gyrus. Age and *cre*-driver are indicated below. Unpaired *t*-test, * = *p* < 0.05, n.s. = not significant, n=3 animals per condition for P0, P14, and 4 months; n=4 animals per condition for 8 month animals. (**B**, **D**) Cryosections of brains were stained for γH2AX as in Fig. 7 and foci counted in the dentate gyrus (**B**) or CA1 layer in 50×40 μm counting frames (**D**). White arrows represent γH2AX foci in the nucleus, yellow arrows represent background staining that was not counted. (**C**, **E**) Quantification of γH2AX in the dentate gyrus (**C**) or CA1 layer (**E**). The *y*-axis is γH2AX foci per counting frame. ANOVA with Tukey's HSD, * = *p* < 0.05. n = 3 animals per experimental condition. Error bars represent s.e.m.

The above data indicates that Cyclin A2 is important in the development of the VZ/SVZ that gives rise to the cerebral cortex. We next asked if Cyclin A2 was necessary for the development of other stem cell niches in the brain. We previously demonstrated that Cyclin A2 is critically important for the development of the cerebellum [3]. Similar to the above results in the VZ/SVZ, growth of the cerebellar EGL stem cell niche was delayed with an increase in apoptosis. However, in contrast to the VZ/SVZ, the cerebellum fails to form appropriately. These contrasting results led us to hypothesize that Cyclin A2 was necessary for the development and function during adulthood of another stem cell niche in the CNS, the dentate gyrus of the hippocampus. To test this hypothesis, we used different *cre* drivers to ablate Cyclin A2 expression in the brain at different times during development. We used *Nestin-cre* for embryonic ablation, and *CamkIIα-cre* for ablation in the adult after the brain was fully developed. The *CamkIIα-cre* mouse was first developed to drive *cre* expression in the CA1 layer of the hippocampus, although it displays widespread expression in the hippocampus and cerebral cortex [32]. We performed unbiased stereological volume measurements of the dentate gyrus as we performed in the VZ/SVZ. We found that embryonic ablation of Cyclin A2 led to a drastic reduction in size of the dentate gyrus immediately after birth (Fig. 8A). Unlike the VZ/SVZ, the size of the dentate gyrus did not recover. However, loss of Cyclin A2 after the brain was fully formed by interbreeding with *CamkIIαcre* mice had no effect on the size of the dentate gyrus at 4 or 8 months after birth (Fig. 8A).

We next asked if, similar to the VZ/SVZ, loss of Cyclin A2 led to decreased γH2AX in the hippocampus. We examined both the dentate gyrus and the CA1 layer of the hippocampus, the original target for *cre* expression in the *CamkIIαcre* mouse. We found no significant change in γH2AX in the dentate gyrus in 4 month old animals (Fig. 8B-C). However, we did observe a decrease in γH2AX foci in the CA1 layer in 4 month old animals, and observed a time-dependent decrease in γH2AX in controls in both hippocampal areas in the controls. This time-dependent decrease in γH2AX was much less pronounced in experimental animals (Fig. 8D-E).

**Figure 9:**
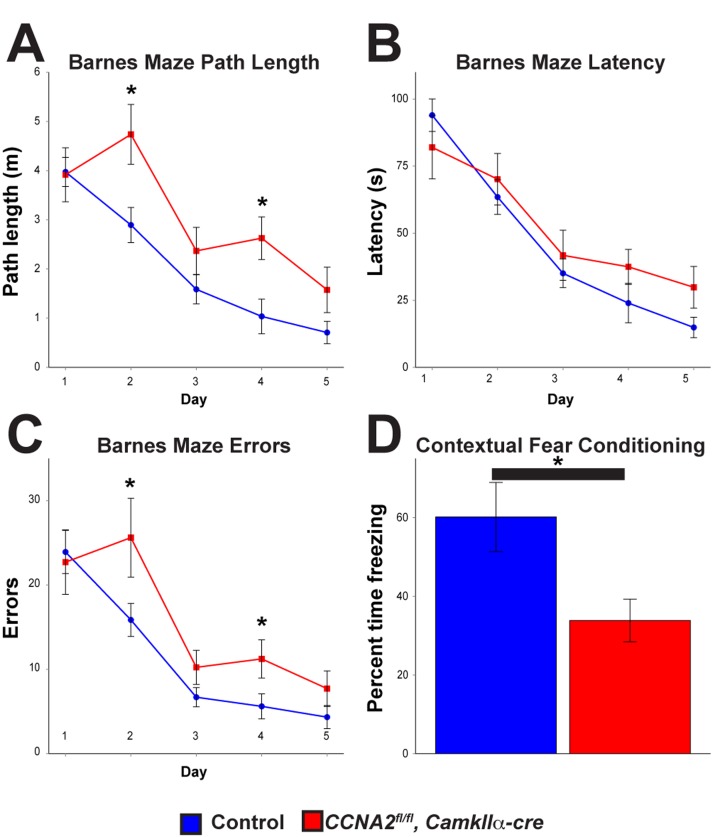
Cyclin A2 loss in the hippocampus leads to deficits in learning and memory *CCNA2^fl/fl^, CamkIIα-cre* mice and control *CCNA2*-intact mice were subjected to a battery of behavioral tests. (**A**-**C**) Barnes maze path length, latency, and errors, respectively. The *x*-axis is testing day. The *y*-axis is path length (**A**), total time to escape (**B**), and total errors during the test (**C**). *CCNA2^fl/fl^, CamkIIα-cre* mice performed worse in the Barnes maze than did control mice. Mixed model ANOVA with post-hoc *t*-test, * = *p* < 0.05. n = 12 control mice and 7 *CCNA2^fl/fl^, CamkIIα-cre* mice. (**D**) Mice were subjected to a fear conditioning test. *CCNA2^fl/fl^, CamkIIα-cre* mice showed less of a response to contextual clues during the test. The *y*-axis is percent of the test time the mouse spent in the “frozen” fear response state. Error bars for all graphs represent s. e. m.

Next, we asked if loss of Cyclin A2 in the developed brain led to appreciable behavioral changes. Based on our γH2AX results in the hippocampus, we hypothesized that these mice would have defects in learning and memory. We therefore subjected 8 month old animals to a battery of behavioral tests. Mice without Cyclin A2 performed significantly worse in the Barnes Maze [33], which tests spatial learning and memory, and fear conditioning [34], which tests contextual memory (Fig. 9A-D). We additionally observed a trend toward decreased performance in the passive avoidance test, although it did not meet our threshold for statistical significance. We did not observe differences in other behavioral tests including anxiety-like responses (open field and elevated plus maze tests), depressive-like responses (forced swim and tail suspension tests), sociability (social preference test), or in marble burying behavior, although there was a small decrease in pain sensitivity and a small increase in Rotarod performance (Fig. Supplemental S3). These results underscore the differential requirement of Cyclin A2 in the development of different structures of the brain. Furthermore, they support a novel role for Cyclin A2 in DNA repair in post-mitotic neurons, which is unexpected given its major role as an S-phase regulator in cycling cells.

## DISCUSSION

### Non-canonical functions of Cyclin A2

DSBs comprise lethal forms of DNA damage, with even a single DSB being capable of killing a cell or disturbing its genomic integrity [35]. The DNA damage response has been well-characterized [36], and the importance of several of these genes in mouse neural development has been described [31, 37-40]. The commonality of these mouse models lies in their hypomorphic cerebellum, which lacks its characteristic lamination and foliation. We previously reported the effect of Cyclin A2 deletion on cerebellar development [3]. Mice without Cyclin A2 during embryonic neural development displays striking similarities to the aforementioned DNA repair mouse mutants, with the same defects in cerebellar morphology. However, we observe a decrease in H2AX phosphorylation, while mutants of many of the other DNA repair genes accumulate γH2AX foci, lending further credence to the hypothesis that Cyclin A2 is a high-level regulator of the DNA damage response.

The most commonly employed DNA repair mechanisms for DSB include homologous re-combination (HR) and non-homologous end joining (NHEJ), and these mechanisms show predilection for distinct cell cycle phases [41]. Specifically, according to this notion NHEJ would be most robust for repair of DNA damage incurred during G1, while HR would be more common for repair during S and G2 [42]. Cyclins and CDKs have been implicated in DSB repair, including Cyclin D1, Cyclin A1, CDK1, and CDK2 [43-45]. Cyclin A2 has previously been shown to interact with both BRCA1 [46] and BRCA2 [47], although to our knowledge we are the first to report that Cyclin A2 is found at the site of DNA damage. It is unexpected however that although it is an S-phase cyclin, Cyclin A2 regulates both HR and NHEJ. This supports our notion that Cyclin A2 is a regulator of DNA damage in post-mitotic neurons, and provides an explanation for our previous finding that Cyclin A2 is expressed in the adult brain [3].

We additionally found that H2AX phosphorylation is inhibited by *CCNA2* loss. These data would indicate that Cyclin A2 lies very early in the DNA repair signal transduction cascade. Further supporting this conclusion are findings that show minimal reduction in HR efficiency by upstream effectors and near complete blockage of HR by downstream effectors of HR. For example, silencing of *ATM*, one of the first genes involved in the DNA damage response results in no significant difference in HR efficiency [48]. In contrast, silencing Rad51 expression results in near complete blockage of HR [43]. These results are more suggestive of a role for Cyclin A2 in the regulation of the DNA damage response rather than in the actual resolution of the DNA double-strand break. Such a notion is in line with known Cyclin A2 functions as a DNA replication fork initiator during S-phase. In S-phase, the chromatin landscape undergoes dramatic alterations as the entire genome is duplicated. Elegantly reviewed by Alabert and Groth, accurate cell cycle progression requires replication initiation at 30,000 to 50,000 origins; no clear DNA consensus sequence for replication origin exists, and it is thought that chromatin structure is a major recognizing factor for replication fork initiation [49]. *CCNA2*'s role as an initiator of replication origin firing [50] thus insinuates a potential role as a chromatin sensor. In summary, our data allows us to conclude that Cyclin A2's well established role as a chromatin sensor may include an ability for this protein to sense DNA double strand breaks.

### Developmental and aging roles for Cyclin A2

Using *CCNA2* ablation as a model, we were able to show cell cycle defects lead to a shift in the quantities of cells produced by the neural stem cell niche. Stated otherwise, the forebrain stem cell niche adapts to cell cycle dysfunction by *inducing a developmental delay,* which allows compensatory accelerated growth mediated by progenitor cells sensing their population size relative to *V_max_*. This delay is well explained by a standard logistic growth mechanism, whereby the rate of population growth depends on the size of the population. This compensation is done at the expense of emerging from the embryonic environment with a decreased computational capacity since the number of neurons generated is lower. The insights obtained from this study carry significant implications for pediatric neurology. Two of the most common environmental fetal genome stressors include alcohol exposure and gestational diabetes. Fetal alcohol exposure induces DNA damage due to aldehyde-mediated DNA cross-linking [51]. Diabetes increases DNA damage, presumably through elevation in reactive oxygen species, sensitizes DNA to damage, and delays DNA repair kinetics [52]. Although the specific chemical changes to DNA mediated by aldehydes and hyperglycemia differ, many types of DNA damaging agents ultimately converge, directly or indirectly, to DNA double-strand break repair [53]. Of particular relevance to our study is the known association of children of gestational diabetic mothers to manifest with *subtle* developmental delays [54, 55]. The implications of our work provide an underlying mechanism as to how fetal stressors, even if transient, can result in long-term developmental delays that manifest months after exposure.

In addition to the role for Cyclin A2 in the repair of DNA damage encountered during neurogenesis, we have further uncovered a role for Cyclin A2 in DNA repair of adult hippocampal neurons. This finding underscores the critical importance of DNA repair in all aspects of life. Indeed, DNA repair malfunction is linked with diseases of the nervous system that are primarily seen early in life such as ataxia-telangiectasia which is caused by mutations in the DNA damage response kinase *ATM* [56]. At the other end of life, many neurodegenerative diseases have been linked with DNA damage. For example, familial amyotrophic lateral sclerosis and frontal temporal lobar degeneration have been linked to mutations in the *FUS* gene which lead to increased DNA damage in neurons [57, 58]. Furthermore, recent evidence has suggested that accumulation of Aβ plaques reduces levels of the well-known DNA repair protein BRCA1 in neurons, leading to the accumulation of DNA damage [59]. Our work demonstrates the differential outcomes resulting from loss of DNA repair function during both development and aging. Supporting this notion are studies on the tumor suppressor *BRCA1* that demonstrated that ablation of *BRCA1* by *Nestin-cre* lead to severe brain growth defects [60]. Additionally, silencing of *BRCA1* in the adult dentate gyrus leads to a similar behavioral phenotype as we observed in our adult-ablated *CCNA2* mice [59]. These studies underscore the importance of the DNA damage response in embryonic neural progenitors as well as in adult neurons.

That Cyclin A2 possesses conserved roles in development and adulthood is at first counter-intuitive. Although the adult brain shows less proliferation than the embryonic brain, the adult brain requires continued cellular turnover in the subgranular zone of hippocampus and the subventricular zone. Furthermore, endogenous DNA damage is an expected finding in both cell types. For example, highly proliferative neural precursor cells in the VZ/SVZ would undergo DNA replication stress, whereas reactive oxygen species-mediated DNA damage represents a well-characterized finding in adult neurons. Nevertheless, we were surprised to find similar phenotypes in DNA damage detection (i.e., a diminished level of endogenous γH2AX in proliferative neural precursor cells and in post-mitotic neurons. We propose that Cyclin A2's function in both homologous recombination and non-homologous end joining-mediated repair permits its role in these two time points. Indeed, the notion of proteins playing conserved roles in both embryonic development and aging has been well-established, and was recently elegantly reviewed by Kovacs, et al. [61]. We submit that through Cyclin A2's non-canonical roles in DNA repair detection, that it has joined the expanding list of genes showing crucial roles in both embryonic development and in the aging brain.

## MATERIALS AND METHODS

### Animal husbandry and tissue processing

All work involving animals was performed under the auspices of a protocol approved by the Ohio State University Institutional Animal Care and Use Committee. *CCNA2^fl/fl^* and *Nestin-cre* transgenic mice were bred as described previously for embryonic ablation of *CCNA2* expression [3]. Briefly, timed-pregnant females were euthanized at embryonic days 14.5 or 17.5, and embryos were drop-fixed in 4% PFA overnight, cryoprotected in 30% sucrose, and snap frozen in OCT. For cell cycle length analyses, an E14.5 pregnant dam was injected with CldU at 50 mg/kg. Embryos were harvested 2.5 hours after CldU administration and drop fixed in 4% PFA. Post-natal day 0 pups were anesthetized with ketamine (30 mg/mL) and xylazine (2 mg/mL) and perfused transcardially with PBS and 4% PFA. P0 pups were pulsed with BrdU for 30 minutes before perfusion as necessary for each experiment. Brains were post-fixed overnight in 4% PFA, cryoprotected in 30% sucrose, and snap frozen in OCT. Cryosections of OCT-embedded brains were taken at 5, 12, or 50 μm and stained with pertinent antibodies. Control mice are littermates of *CCNA2^fl/fl^*, *Nestin-cre* mice with preserved *CCNA2*.

*CCNA2^fl/fl^* and *CamkIIα-cre* transgenic mice were intercrossed for adult ablation of *CCNA2* in the hippocampus and cerebral cortex. 4 month old mice were perfused as above. Brains were bisected sagittally and processed for unbiased stereology cresyl violet staining or OCT embedding. 8 month old mice were subjected to a battery of behavioral tests, then sacrificed and brains processed. Control mice were littermates of *CCNA2^fl/fl^*, *CamkIIαcre* mice with preserved *CCNA2*.

### Unbiased stereology

#### Optical Fractionator

For estimation of cleaved caspase 3-positive cells in the VZ/SVZ, 50 μm cryosections of E14.5 whole embryos or E17.5 brains were used. For estimation of NeuN-positive cells, P0 brains were used. All analyses were performed with a Carl Zeiss Imager M.2 microscope with motorized stage and StereoInvestigator™ software (MBF Biosciences) as described previously by our group [62]. Cleaved caspase 3 analysis used one section of every five and NeuN analysis used one section of every 10 through the entire brain. Optical Fractionator analyses used a 100x oil immersion objective, dissector height of 20 μm, dissector volume of 50,000 μm^3^, counting frame height and width of 50 μm, and sampling grid dimensions of 153.9 × 162.5 μm.

#### Cavalieri estimation

For Cavalieri volume estimation of the VZ/SVZ, 50 μm cryosections of E14.5 whole embryos or E17.5 brains were used. One section of every five through the entire brain was evaluated with Cavalieri probes with a 4x objective with grid size of 30 μm and shape factor of 4. For Cavalieri volume estimation of the dentate gyrus, 50 μm cryosections of P0 brains were stained with hematoxylin, and 100 μm sections of P14, 4 month old, and 8 month old brains were stained with cresyl violet. One section of every ten for P0 brains and one section of every five for P14, 4 month old, and 8 month old brains through the entire brain was evaluated with Cavalieri probes with a 4x objective with grid size of 30 μm and shape factor of 4.

### Derivation of mathematical model

Unbiased stereological* values were used to guide the development of a mathematical model of forebrain development [63]. While a natural description of proliferative populations would be through continuous time stochastic processes such as branching processes or birth-death processes [64-67], our main interest is in the situation where population size is already exponentially high, making any stochastic effects mathematically insignificant. For this reason, we formulated our model through deterministic ordinary differential equations*. Note however that these equations can be interpreted as a mean-field approximation of an associated stochastic branching process.

Radial glia cells in embryonic mice are known to undergo three types of division [1, 68]. The first is proliferation, a type of symmetric self-renewal, where two radial glia daughter cells are produced. The second is an asymmetric self-renewal where a migratory neuron is produced and a radial glial cell is renewed. The third is a different type of asymmetric division where a neuron is produced along with an intermediate progenitor cell in the SVZ, and the radial glia does not undergo self-renewal. The intermediate progenitor undergoes another division to yield two migratory neurons. We incorporated all of these types of division into a mathematical model. The number of cells present in each of the stages is given by a system of ordinary differential equations presented in Supplemental Tables S2 and S3.

The terms *J*_(·)_ denote rates of exit from each of the states and are denoted graphically in Fig. 2C and in Supplemental Table S4. These flows can be understood in terms of cycle transition times on the cell-by-cell basis. They are parameterized* by transition rates, which are related to the mean times taken for cells to progress between the different stages in cell cycle. In turn, the timing is determined by the detailed biochemistry of the cells under influence from both intracellular and extracellular environments. While the precise biochemistry with respect to changes in Cyclin A2 expression is not known with certainty, we were still able to investigate the effects of Cyclin A2 on development by examining how the growth of the VZ/SVZ is perturbed. We performed this task by first parameterizing our model using experimental observations found in the literature. Prior researchers have reported the timing of the various cell-cycle transitions in wild-type mice in the embryonic ages. We used this timing information to deduce equivalent rates for the transitions (shown in Supplemental Tables S4 and S5).

The transitions in this model generally follow first-order kinetics. The exceptions are the transition rates out of G1 for the radial glia cells. G1 phase is known to lengthen in the course of the development of the VZ/SVZ, concomitant with a shift towards the production of differentiated cells and away from proliferative precursors [15]. We take this fact to imply that the effective average G1 cycle length can be given as

$$t_{G1}=pt_{G1P}+(1-p-q)t_{G1R}+{\mathrm{qt}}_{G1D}$$

where *t*_*G*1*D*_ is the G1 cycle length for asymmetric division into differentiated cells, *t*_*G*1*P*_ is the G1 cycle length for proliferation of the neural stem cells, and *t*_*G*1*R*_ is the G1 cycle length for asymmetric divisions of the radial glia that are associated with self-renewal. The terms *p*, *q* describe the relative proportion of each of the different types of mitosis. As developmental age increases, and the number or density of cells increases, there is a transition from proliferative-type divisions to renewal to differentiation (neurogenesis). The biological basis for these transitions from proliferation to self-renewal and neurogenesis is complicated, involving both intrinsic and extrinsic signaling factors, which change temporally. These factors include the developmental age of the cells, presence and activation of cell-surface signaling proteins such as αE-catenin [69], Notch [70], and Jagged [71], as well as diffusing growth factors like HH amongst others.

In order to determine when differentiation occurs, we utilized the data of Takahashi et al. [15], where the authors reported the fraction of cells exiting cell cycle into neurogenesis as a function of developmental age (e.g., a cell of age one divides into two cells of age two). We interpreted these data as a survival probability for the mitotic capability of the progenitors, where we assume that progenitors exit cell cycle by asymmetrically differentiating into an intermediate progenitor and a neuron. In our model we assume that radial glia differentiate when they reach a preset number of divisions *K*. In our model, wildtype radial glia have K=5 available divisions at E13.5, which corresponds to approximately 2 divisions at E14.5.

We assume that a separate biochemical switch that depends on interactions with other radial glia determines the proportion of proliferative cells *p* versus self-renewing cells. We model this switch by assuming that *p* can be described as a function of the population size. We suspect on the basis of our data that this is so, as growth is slower in the wildtype VZ/SVZ in the days between E14.5 and E17.5 compared to in the mutant, which trails the wildtype in VZ/SVZ volume as well as CP volume. We postulate that this difference in growth rates can be due to the decreased inhibition of mutant growth, which can be explained through a logistic growth mechanism. The logistic growth model for population dynamics assumes that the overall growth rate is proportional to the size of the population and the amount of available resources (in our case, space, as well as growth factors) [72]. It has also previously been noted that the brain appears to develop according to a logistic growth curve [73]. We model logistic growth by setting *p* using the expression

$$p=1-\frac{V_{VZ}}{V_{\text{max}}}$$

where the quantity *V*_*max*_ is the effective carrying capacity of the VZ. The model is assumed to start at day E13.5 with initial conditions given to yield the total cumulative volume observed in the data at E14.5. We assumed that initially all cells in the simulation are of the same mitotic age, and that the proportion of cells in each of the phases of cell cycle is weighted by the time spent in each phase.

The growth of the cortical plate depends heavily on the migration parameter contained in the migration flow *J*_*migrate*_. The latency involved in migration of the differentiated neuron to the cortical plate depends on the changing dimensions of the developing cortex. As the distance between the SVZ and the CP increases, it is expected that neurons take longer to reach the CP. Understanding the kinetics of this process, while interesting in itself, is outside of the scope of the questions that we hope to answer with this model. For this reason, we report the cumulative output of differentiated neurons from the VZ/SVZ rather than the volume of the cortical plate. Yet, we retain this migration in the model to emphasize that the dynamics of the cortical plate volume can be computed provided one has a description of the migration rate.

### Cell culture and viral infections

All cell culture was performed in a 37°C humidified incubator set at 5% CO_2_ in room air. U251 glioblastoma cells were cultured in DMEM/F12 supplemented with non-essential amino acids and glutamine with 10% fetal bovine serum. Previously published and experimentally-validated *CCNA2*-targeting or scrambled non-targeting shRNA lentiviral constructs were a kind gift of Dr. Benedicte Lemmers and Dr. Jean-Marie Blanchard and were produced as described [23]. U251 cells were transduced with lentivirus encoding either *CCNA2*-targeting or scrambled shRNA. Transduced cells were selected by culturing in medium containing 1.0 μg/mL puromycin, and maintained in puromycin medium for all experiments. Cells were irradiated with 160 kV X-rays using a RadSource RS2000 Biological Irradiator at the appropriate doses for each experiment. Cells were fixed with 4% PFA before immunocytochemical processing. To generate *CCNA2^fl/fl^* neurospheres, *CCNA2^fl/fl^* males were intercrossed with *CCNA2^fl/fl^* females to generate homozygous pups. Postnatal neural progenitor cultures were generated by dissecting the VZ/SVZ from brain, and cultured in suspension with DMEM/F12 media supplemented by bFGF/EGF, N2, B27, glutamax, penicillin/streptomycin, and heparin. For the proliferation assays, neurospheres were dissociated with trypsin to single cells and plated onto PDL/laminin coated chamber slides followed by infection with 1 μl adenovirus (adenovirus-*GFP* for control, adenovirus-*cre-GFP* as the experimental group, Vector Biolabs). Cre-mediated excision was confirmed by amplification of genomic DNA with the following primers: LoxP1 #1a: 5′-CGCAGCAGAAGCTCAAGACTCGAC-3′; LoxP1 #2: 5′-TCTACATCCTAATGCAATGCCTGG-3′; LoxP2 4a: 5′-TGTACAGCATGGACTCCGAGCGAC-3′. Cells were pulsed with BrdU for 30 minutes, fixed, and immunostained for BrdU and pH3.

### Proximity ligation assay

Fixed cells were per-meabilized for 5 minutes with PBS containing 0.1% Triton X-100, then blocked with blocking solution (PBS with 0.1% Triton X-100 and 5% normal goat serum) for 30 minutes. Cells were incubated overnight at 4°C with primary antibodies against Cyclin A2 and γH2AX diluted in blocking solution. Control cells were single-labeled with either Cyclin A2 or γH2AX antibody. Coverslips were then washed 3×5 minutes with 3 mL PBS. PLA was performed according to manufacturer's instructions (O-link Biosciences). Maximum intensity projection images from .czi files were thresholded and number of foci in each nucleus was automatically counted using ImageJ.

### Comet assay

Cells were irradiated at 2 Gy. Control cells were not irradiated. Cells were dissociated with accutase, quenched with cold medium, and harvested by centrifugation. Cell pellets were washed with ice-cold PBS, and prepared for comet assays. Cells were mixed with 1% low-melting point (LMP) agarose, and 100 μL of cells in agarose was pipetted onto an agarose-coated slide and covered with a coverglass. Slides were chilled on an ice-cold aluminum plate for 10 minutes and coverglass was removed. The cell-agarose mixture was covered with 1% LMP agarose and covered with a coverglass. Slides were chilled as before. Coverslips were removed, and slides were processed as described previously [74]. Slides were stained with ethidium bromide, visualized at 20x magnification, and scored using OpenComet software [75].

### Homologous recombination and non-homologous end joining assays

Plasmids pDRGFP (Addgene #26475) and pCBASceI (Addgene #26477) were obtained from Maria Jasin via the Addgene repository [24]. PimEJ5GFP (Addgene #44026) was obtained from Jeremy Stark via the Addgene repository [25]. U251 cells were transfected with pDRGFP or pimEJ5GFP, and cells with the integrated plasmids were selected with medium containing puromycin at a concentration of 1.0 μg/mL and expanded for subsequent experiments. To measure levels of homologous recombination or non-homologous end joining, cells were plated in two 6-well plates at a concentration of 150,000 cells/well. Cells were transfected overnight with either *CCNA2* siRNA (Invitrogen siRNA #4390824) or control siRNA #1 (Invitrogen catalog #4390843) at a concentration of 10 nM with JetPrime (Polyplus Transfection) following the manufacturer's protocol. Following transfection, cells were re-plated at a density of 150,000 cells/well in 6-well plates and incubated overnight. Cells were then transfected overnight with *CCNA2* or control siRNA together with 2 μg pCBASceI plasmid to induce a double-strand break at the DRGFP or EJ5GFP locus. Parallel controls were performed with pCAGGS for gating of cells for flow cytometry. DRGFP cells were re-fed with fresh puromycin medium every other day for 6 days before dissociation with accutase. EJ5GFP cells were refed with fresh medium without puromycin every other day for 5 days before dissociation with accutase. GFP+ cells were quantified using a Becton-Dickinson FACSCalibur flow cytometer. The threshold for GFP-negative cells was set using pCAGGS-transfected cells, and cells with fluorescence above this threshold were counted as GFP-positive. Gating strategy is shown in Fig. Supplemental S2. 20,000 cells were analyzed, and percentage of GFP-positive cells was determined relative to the total population. Relative levels of HR or NHEJ were determined by normalizing the percentage of GFP-positive cells in each condition to the percentage of GFP-positive cells in control conditions transfected with control siRNA and pCBASceI plasmid.

### Clonogenic assay

U251 cells infected with lentivirus expressing shRNA targeting *CCNA2* or non-targeting scrambled shRNA were seeded at clonal densities in 6 well plates and allowed to adhere for 1 hour at 37°C. Cells were then irradiated at 0, 3, 6, or 9 Gy at room temperature, and allowed to recover and grow for approximately 1 week in the cell culture incubator. Cells were re-fed with fresh medium every other day. After growth, cells were fixed for 15 minutes in 4% PFA, stained for 30 minutes with 0.05% crystal violet, washed with water, and dried. Colonies were manually counted on a Zeiss Discovery V.8 Stereomicroscope, and surviving fraction (SF) determined for each dose using the following formula: SF=#cells counted/(#cells plated * plating efficiency).

### Western blotting

Cells were lysed in lysis buffer containing 50 mM Tris-HCl, pH 6.8, 1% SDS, 1 mM EDTA, 5% glycerol, and 1x Halt protease/phosphatase inhibitor (Pierce). Total cell lysates from U251 *CCNA2*-knockdown or scrambled cells, or U251 *CCNA2* or control siRNA-transfected cells were resolved on an SDS-PAGE gel and transferred to a nitrocellulose membrane. For determination of *CCNA2* silencing, membranes were probed with antibodies against Cyclin A2 and GAPDH as a loading control. Blots were visualized using Cy3 or Cy5 ECL-Plex secondary antibodies with a Typhoon scanner or with HRP-conjugated secondary antibodies with a Bio-Rad Chemidoc XRS. Quantification of Western blot images was performed by densitometric analysis using ImageJ.

### Antibodies and histology

Antibodies used in this study for immunohistochemistry (IHC) and western blotting (WB) were as follows: BrdU, GeneTex #gtx26326, IHC 1:600 Cleaved caspase 3, Cell Signaling Technologies #9664S, IHC 1:100 dilution; CldU, Novus Biologicals # NB500-169 IHC 1:600 dilution; Cyclin A2, Santa Cruz Biotechnology #sc-596 IHC 1:100, WB 1:1000; GAPDH, Millipore #MAB374, WB 1:1000; γH2AX, Millipore #05-636, IHC 1:200; γH2AX, Cell Signaling Technologies #9718, IHC 1:400; NeuN, Millipore #MAB377, IHC 1:1000; Pax6, Santa Cruz Biotechnology #sc-7750 IHC 1:100 or DSHB #PAX6 IHC 1:5; pH3, Cell Signaling Technologies #2650, IHC 1:100; Rad51, Calbiochem #PC130, 1:200 for IHC on tissue, 1:300 for cell culture; Tbr2 was a kind gift from Dr. Robert Hevner and was used at 1:500 dilution for IHC. Alexa Fluor-conjugated secondary antibodies were from Invitrogen and were used at a 1:1000 dilution.

For unbiased stereology analyses of E14.5 and E17.5 embryos, 50 μm cryosections were labeled with antibodies raised against cleaved caspase 3. For unbiased stereology analyses of P0 brains, 50 μm cryosections were labeled with antibodies raised against NeuN. Stains were developed with the Dako EnVision HRP kit. Nuclei were counterstained with hematoxylin and tissue sections were dehydrated for 2 minutes each in 10%, 20%, 40%, 80%, 95%, and twice in 100% dehydrant. Sections were incubated for 1 minute each in sequential xylene baths, and coverslips were mounted with Permount.

For immunofluorescent analysis, tissue sections were subjected to antigen retrieval with 10 mM citrate buffer pH 6.0 in a pressure cooker for 4 minutes or Pelco BioWave at 95°C for 15 minutes, blocked with blocking solution, and labeled with the appropriate primary antibodies for each experiment. Sections were washed with PBS and incubated with the appropriate secondary antibodies and counterstained with DAPI. Sections were washed and coverslips mounted with Prolong Gold (Invitrogen). Cultured cells were processed in a similar fashion without antigen retrieval.

### Microscopy and imaging

Fluorescent images were captured with a Carl Zeiss Axio Imager Z.1 with LSM700 confocal laser scanning microscope with 10x, 20x, or 63x objectives as necessary, or with a Nikon 80i epifluorescent microscope with Hamatasu Orca-R2 camera. For analysis of total Rad51-positive cells, single optical slice images or Z-stacks were captured at 200x magnification. For analysis of γH2AX in tissue sections, Z-stacks with step size 0.38 μm encompassing the entire thickness of the histological preparation were acquired at 630x magnification. For analysis of γH2AX or PLA foci in cultured cells, Z-stacks encompassing the entire thickness of the nucleus were acquired. All Z-stack analyses for cultured cells used a 63x objective with a pinhole of 1 Airy unit, scan speed of 6, averaging of 1, and resolution of 512×512. Maximal projections of Z-stacks were analyzed in ImageJ to count foci or proportion of the nucleus positive for each marker. For analysis of Cyclin A2, Pax6, Tbr2, and CldU staining, images of the dorsal cortex were captured at 100x or 200x magnification. 100x images were assembled as a montage and placed on a black background for visual clarity. 200x images were captured as Z-stacks in .czi files and converted to .tif images. Images were counted in a standard counting frame (100 μm medial-lateral, 180 μm dorsal-ventral starting at the boundary of the lateral ventricle) on the dorsal cortex and when necessary divided into 100 × 10 μm bins as described previously [76]. Counts for Tbr2 utilized total counts within the entire counting frame. To account for the decreased size of the brain, Tbr2 counts were multiplied by the size of the VZ/SVZ obtained by unbiased stereology measurements. Counts within the first bin along the ventricle wall were used for CldU quantification. To overcome the limitations of quantifications of Rad51 foci in tissue sections, we instead performed fluorescent intensity measurements in E14.5 animals or counted proportion of Rad51-positive cells in P0 animals. To achieve unbiased quantifications of γH2AX in neuroanatomical structures, we used a similar methodology to count foci in counting frames rather than in individual cells. Quantification of γH2AX in E14.5 embryos was performed using 630x magnification images with a standard counting frame of 50 μm medial-lateral × 90 μm dorsal-ventral to include the VZ/SVZ. Quantification of γH2AX in 4 month old and 8 month old brains was performed using 630x magnification images with a standard counting frame of 50 μm medial-lateral × 40 μm dorsal-ventral to include the dentate gyrus or CA1 layer. For γH2AX counting, nuclear signals smaller than 0.8 μm diameter were counted [77]. We have extensive experience quantifying γH2AX in the brain, and note that there is background signal that is generally extra-nuclear. To avoid parameterizing the data, we chose to count events within a nucleus in a defined area to maintain a normal distribution. All images were acquired as greyscale images and presented as pseudocolored images.

### Behavioral testing

All behavioral testing was performed by the Ohio State University Rodent Behavioral Core Facility. *CCNA2^fl/fl^*, *CamkIIα-cre* mice and control littermates with preserved *CCNA2* were subjected to the following battery of behavioral tests. Twelve controls and seven *CCNA2^fl/fl^*, *CamkIIα-cre* mice of mixed sex were used.

#### Barnes maze

The Barnes maze was performed as described previously [78]. Mice were placed on a 91 cm diameter white circular polypropylene disk elevated 1.2 meters above the floor. The disk had eighteen 5 cm diameter holes spaced around the perimeter with one hole leading to an escape box (San Diego Instruments, CA). Noldus tracking software (Leesburg, VA) was used to record latency to reach the escape hole, path length, and number of errors. Testing occurred in a brightly lit room between one and six hours after the onset of the light phase. Mice were trained for five days with three trials per day. The intertribal interval was approximately 10 min. If the mouse did not find the hole within the first two minutes of a trial, then the latency was recorded as 120 seconds and the mouse was guided to the escape hole.

#### Fear conditioning test

The fear conditioning test was performed similarly to that described previously [34]. Mice were placed in a plexiglass box with a metal grid floor, which is used to administer a foot shock (MedAssociates, Georgia, VT). This apparatus is inside a light- and sound-attenuating chamber with a video camera on the door to record freezing activity of the mouse using VideoFreeze (MedAssociates). A background noise of 68dB was played during Sessions 1 and 2. Session 1 (Day1, acclimation): Mice were acclimated for 180 seconds, then played an 80dB tone for 20sec, followed by a 0.6mA shock during the final second of the tone. The tone and shock was administered 7 times with a 30 second intertribal interval. Mice were returned to their home cage after a 60 second final observation period. Session 2 (Day 2, contextual): Approximately 24h after the first session, each mouse was placed in the box and freezing activity was recorded for 180 sec. Mice were then placed back in their home cages. Session 3 (Day 2 evening, cued): This session was performed as in Session 1, except no shock was administered. Furthermore, the environment was altered by addition of vanilla or peppermint scent, addition of a white floor panel and insert to change the shape of the box, addition of construction paper on the inside of the chamber wells, and a black cover over the box to isolate tone-dependent memory. Animals were returned to their cages following the test.

#### Rotarod test

The rotarod test was performed as described previously [79]. Each mouse was placed on a rotating rod (Med Associates, Inc.) for three separate trials. The rod was accelerated starting at 4 revolutions per minute (RPM) and ending at 40 RPM The time until the mouse fell off the rotarod was recorded over three trials and averaged.

#### Hot plate test

The hot plate test was performed as described previously [80]. Mice were placed on a 51°Celsius hot plate and pain response measured by time until the mouse licked its paw. Each mouse was evaluated for time to response in two separate trials.

#### Grip strength

Grip strength test was performed similarly to that described previously [81]. Mice were placed on a wire grid attached to a force gauge (Chatillon Ametek, Inc.). Each mouse was pulled gently by its tail until it released from the grid, and the force required was measured. Each mouse was subjected to three trials.

#### Olfactory test

Olfactory test was performed as described previously [82]. Mice were food-restricted for 1 day, and placed in a cage with food buried in the bedding. Mice were scored for the latency to find and uncover the hidden food.

#### Social preference test

Mice were placed in an apparatus consisting of 3 connected Plexiglass chambers with removable dividers between each chamber. Each mouse was placed in the central chamber for 5 minutes of habituation. An ovariectomized female was then placed in one of the side chambers in a wire cage and the dividers were lifted for a 10 minutes period. Mice were video recorded and the time spent in each testing chamber was measured.

#### Open field test

Open field test was performed as described previously [83]. Each animal is placed in an Open Field Photobeam Activity System (San Diego Instruments, Inc) in a light- and sound-attenuated box. The arenas are contained in boxes that are light- and sound-attenuating. Amount of activity in the center versus the periphery of the arena was analyzed.

#### Elevated plus maze test

The elevated plus maze was performed as described previously [84]. Animals were tested during the early dark phase (0-2hr after lights off). Mice were placed in the center of the platform facing a closed arm, and scored for time in center, time spent in open arms, and time spent in closed arms over a 5 minute duration.

#### Passive avoidance test

Passive avoidance test was performed similarly to that described previously [85]. Mice were placed in the Gemini Avoidance System (San Diego Instruments, Inc.), consisting of 2 darkened chambers with a wire grid floor, separated by a vertical stainless steel wall with an automatic doorway. Mice were placed inside the lighted chamber in a clear acrylic box. One side of the box is open against a closed doorway between the two chambers. After 30 seconds, the door is opened and latency to enter the darkened chamber is recorded. When the mouse entered the chamber, an electrical current of 0.6 milliamps was delivered to the mouse via the grid floor. The duration of the shock was 2 sec. Total test time was 300 seconds. The test was repeated 24 hours later and the difference in latency recorded.

#### Marble burying test

The marble burying test was performed as described [86]. Mice were placed in cages filled with 5 cm of cedar bedding. Fifteen marbles were placed in a regular pattern on the bedding, and each mouse was placed in the test chamber for 20 minutes. Marbles were scored as “buried” when they were embedded more than 2/3 their depth in the bedding. Total number of unburied marbles was evaluated for each mouse.

#### Porsolt forced swim test

Porsolt forced swim test was performed as previously described [87]. Mice were placed in a 24 cm diameter glass cylinder filled to a depth of 14 cm with 26°C water for 5 minutes. Mice were videotaped and analyzed time spent immobile versus swimming. Mice were dried under a heat lamp for 5 minutes then returned to their cages.

#### Tail suspension test

The tail suspension test was performed as described [88]. Mice were suspended by the tip of the tail using laboratory tape. Behavior was documented through video recording for 6 min, and scored for time spent immobile versus active. Mice that were able to climb up their tail were excluded from the analysis.

### Statistical analysis

Descriptive and inferential statistics were performed in Microsoft Excel or R v.3.0.1. Student's *t-*test for pairwise comparison or ANOVA with Tukey's HSD correction for multiple comparisons were used as appropriate for each experiment as indicated in figure legends.

## SUPPLEMENTAL DATA FIGURES AND TABLES



## Footnotes

1 We have included a glossary to define terms related to the mathematical model presented herein. Such terms are denoted by an asterisk and are defined in the supplemental materials.

## References

[1] Lui JH, Hansen DV, Kriegstein AR (2011). Development and evolution of the human neocortex. Cell.

[2] Hatten ME, Heintz N (1995). Mechanisms of neural patterning and specification in the developing cerebellum. Annu Rev Neurosci.

[3] Otero JJ, Kalaszczynska I, Michowski W, Wong M, Gygli PE, Gokozan HN, Griveau A, Odajima J, Czeisler C, Catacutan FP, Murnen A, Schuller U, Sicinski P (2014). Cerebellar cortical lamination and foliation require cyclin A2. Dev Biol.

[4] May RM (1976). Simple mathematical models with very complicated dynamics. Nature.

[5] Keyfitz N, Campbell DM, Higgins JC (1984). Mathematics and Population. Mathematics: People, Problems, Results.

[6] Edelstein-Keshet L (2005). Mathematical models in biology.

[7] Takahashi T, Nowakowski RS, Caviness VS (1993). Cell cycle parameters and patterns of nuclear movement in the neocortical proliferative zone of the fetal mouse. J Neurosci.

[8] Takahashi T, Nowakowski RS, Caviness VS (1995). The cell cycle of the pseudostratified ventricular epithelium of the embryonic murine cerebral wall. J Neurosci.

[9] Takahashi T, Nowakowski RS, Caviness VS (1997). The mathematics of neocortical neuronogenesis. Dev Neurosci.

[10] Gohlke JM, Griffith WC, Faustman EM (2004). The role of cell death during neocortical neurogenesis and synaptogenesis: implications from a computational model for the rat and mouse. Brain research Developmental brain research.

[11] Barton A, Fendrik AJ, Rotondo E (2014). A stochastic model of neurogenesis controlled by a single factor. J Theor Biol.

[12] Noctor SC, Martinez-Cerdeno V, Ivic L, Kriegstein AR (2004). Cortical neurons arise in symmetric and asymmetric division zones and migrate through specific phases. Nat Neurosci.

[13] Miyata T, Kawaguchi A, Saito K, Kawano M, Muto T, Ogawa M (2004). Asymmetric production of surface-dividing and non-surface-dividing cortical progenitor cells. Development.

[14] Noctor SC, Martinez-Cerdeno V, Kriegstein AR (2008). Distinct behaviors of neural stem and progenitor cells underlie cortical neurogenesis. J Comp Neurol.

[15] Takahashi T, Nowakowski RS, Caviness VS (1996). The leaving or Q fraction of the murine cerebral proliferative epithelium: a general model of neocortical neuronogenesis. J Neurosci.

[16] Conrad MS, Dilger RN, Johnson RW (2012). Brain growth of the domestic pig (Sus scrofa) from 2 to 24 weeks of age: a longitudinal MRI study. Dev Neurosci.

[17] Kalaszczynska I, Geng Y, Iino T, Mizuno S, Choi Y, Kondratiuk I, Silver DP, Wolgemuth DJ, Akashi K, Sicinski P (2009). Cyclin A is redundant in fibroblasts but essential in hematopoietic and embryonic stem cells. Cell.

[18] Zimmerman L, Parr B, Lendahl U, Cunningham M, McKay R, Gavin B, Mann J, Vassileva G, McMahon A (1994). Independent regulatory elements in the nestin gene direct transgene expression to neural stem cells or muscle precursors. Neuron.

[19] Englund C, Fink A, Lau C, Pham D, Daza RA, Bulfone A, Kowalczyk T, Hevner RF (2005). Pax6, Tbr2, and Tbr1 are expressed sequentially by radial glia, intermediate progenitor cells, and postmitotic neurons in developing neocortex. J Neurosci.

[20] Goldstone S, Pavey S, Forrest A, Sinnamon J, Gabrielli B (2001). Cdc25-dependent activation of cyclin A/cdk2 is blocked in G2 phase arrested cells independently of ATM/ATR. Oncogene.

[21] Soderberg O, Gullberg M, Jarvius M, Ridderstrale K, Leuchowius KJ, Jarvius J, Wester K, Hydbring P, Bahram F, Larsson LG, Landegren U (2006). Direct observation of individual endogenous protein complexes in situ by proximity ligation. Nat Methods.

[22] Lobrich M, Shibata A, Beucher A, Fisher A, Ensminger M, Goodarzi AA, Barton O, Jeggo PA (2010). gammaH2AX foci analysis for monitoring DNA double-strand break repair: strengths, limitations and optimization. Cell Cycle.

[23] Arsic N, Bendris N, Peter M, Begon-Pescia C, Rebouissou C, Gadea G, Bouquier N, Bibeau F, Lemmers B, Blanchard JM (2012). A novel function for Cyclin A2: control of cell invasion via RhoA signaling. J Cell Biol.

[24] Pierce AJ, Johnson RD, Thompson LH, Jasin M (1999). XRCC3 promotes homology-directed repair of DNA damage in mammalian cells. Genes Dev.

[25] Bennardo N, Cheng A, Huang N, Stark JM (2008). Alternative-NHEJ is a mechanistically distinct pathway of mammalian chromosome break repair. PLoS Genet.

[26] Henriksson S, Rassoolzadeh H, Hedstrom E, Coucoravas C, Julner A, Goldstein M, Imreh G, Zhivotovsky B, Kastan MB, Helleday T, Farnebo M (2014). The scaffold protein WRAP53beta orchestrates the ubiquitin response critical for DNA double-strand break repair. Genes Dev.

[27] Franken NA, Rodermond HM, Stap J, Haveman J, van Bree C (2006). Clonogenic assay of cells in vitro. Nat Protoc.

[28] Allen C, Ashley AK, Hromas R, Nickoloff JA (2011). More forks on the road to replication stress recovery. J Mol Cell Biol.

[29] Cooke MS, Evans MD, Dizdaroglu M, Lunec J (2003). Oxidative DNA damage: mechanisms, mutation, and disease. FASEB J.

[30] Cohen Y, Dardalhon M, Averbeck D (2002). Homologous recombination is essential for RAD51 up-regulation in Saccharomyces cerevisiae following DNA crosslinking damage. Nucleic Acids Res.

[31] Rodrigues PM, Grigaravicius P, Remus M, Cavalheiro GR, Gomes AL, Martins MR, Frappart L, Reuss D, McKinnon PJ, von Deimling A, Martins RA, Frappart PO (2013). Nbn and atm cooperate in a tissue and developmental stage-specific manner to prevent double strand breaks and apoptosis in developing brain and eye. PLoS One.

[32] Tsien JZ, Chen DF, Gerber D, Tom C, Mercer EH, Anderson DJ, Mayford M, Kandel ER, Tonegawa S (1996). Subregion- and cell type-restricted gene knockout in mouse brain. Cell.

[33] Barnes CA (1979). Memory deficits associated with senescence: a neurophysiological and behavioral study in the rat. J Comp Physiol Psychol.

[34] Curzon P, Rustay NR, Browman KE, Buccafusco JJ (2009). Cued and Contextual Fear Conditioning for Rodents. Methods of Behavior Analysis in Neuroscience.

[35] Frankenber-Schwager M, Frankenberg D (1990). DNA double-strand breaks: their repair and relationship to cell killing in yeast. International Journal of radiation biology.

[36] Ciccia A, Elledge SJ (2010). The DNA damage response: making it safe to play with knives. Mol Cell.

[37] Frappart PO, Tong WM, Demuth I, Radovanovic I, Herceg Z, Aguzzi A, Digweed M, Wang ZQ (2005). An essential function for NBS1 in the prevention of ataxia and cerebellar defects. Nat Med.

[38] Ciemerych MA, Kenney AM, Sicinska E, Kalaszczynska I, Bronson RT, Rowitch DH, Gardner H, Sicinski P (2002). Development of mice expressing a single D-type cyclin. Genes Dev.

[39] Lee Y, Katyal S, Downing SM, Zhao J, Russell HR, McKinnon PJ (2012). Neurogenesis requires TopBP1 to prevent catastrophic replicative DNA damage in early progenitors. Nat Neurosci.

[40] Lee Y, Shull ER, Frappart PO, Katyal S, Enriquez-Rios V, Zhao J, Russell HR, Brown EJ, McKinnon PJ (2012). ATR maintains select progenitors during nervous system development. EMBO J.

[41] Ferreira MG, Cooper JP (2004). Two modes of DNA double-strand break repair are reciprocally regulated through the fission yeast cell cycle. Genes Dev.

[42] Takata M, Sasaki MS, Sonoda E, Morrison C, Hashimoto M, Utsumi H, Yamaguchi-Iwai Y, Shinohara A, Takeda S (1998). Homologous recombination and non-homologous end-joining pathways of DNA double-strand break repair have overlapping roles in the maintenance of chromosomal integrity in vertebrate cells. The EMBO journal.

[43] Jirawatnotai S, Hu Y, Michowski W, Elias JE, Becks L, Bienvenu F, Zagozdzon A, Goswami T, Wang YE, Clark AB, Kunkel TA, van Harn T, Xia B (2011). A function for cyclin D1 in DNA repair uncovered by protein interactome analyses in human cancers. Nature.

[44] Manfrini N, Guerini I, Citterio A, Lucchini G, Longhese MP (2010). Processing of meiotic DNA double strand breaks requires cyclin-dependent kinase and multiple nucleases. The Journal of biological chemistry.

[45] Federico M, Symonds CE, Bagella L, Rizzolio F, Fanale D, Russo A, Giordano A (2010). R-Roscovitine (Seliciclib) prevents DNA damage-induced cyclin A1 upregulation and hinders non-homologous end-joining (NHEJ) DNA repair. Mol Cancer.

[46] Ruffner H, Jiang W, Craig AG, Hunter T, Verma IM (1999). BRCA1 is phosphorylated at serine 1497 in vivo at a cyclin-dependent kinase 2 phosphorylation site. Mol Cell Biol.

[47] Esashi F, Christ N, Gannon J, Liu Y, Hunt T, Jasin M, West SC (2005). CDK-dependent phosphorylation of BRCA2 as a regulatory mechanism for recombinational repair. Nature.

[48] Kass EM, Helgadottir HR, Chen CC, Barbera M, Wang R, Westermark UK, Ludwig T, Moynahan ME, Jasin M (2013). Double-strand break repair by homologous recombination in primary mouse somatic cells requires BRCA1 but not the ATM kinase. Proc Natl Acad Sci U S A.

[49] Alabert C, Groth A (2012). Chromatin replication and epigenome maintenance. Nat Rev Mol Cell Biol.

[50] Katsuno Y, Suzuki A, Sugimura K, Okumura K, Zineldeen DH, Shimada M, Niida H, Mizuno T, Hanaoka F, Nakanishi M (2009). Cyclin A-Cdk1 regulates the origin firing program in mammalian cells. Proc Natl Acad Sci U S A.

[51] Oberbeck N, Langevin F, King G, de Wind N, Crossan GP, Patel KJ (2014). Maternal aldehyde elimination during pregnancy preserves the fetal genome. Mol Cell.

[52] Blasiak J, Arabski M, Krupa R, Wozniak K, Zadrozny M, Kasznicki J, Zurawska M, Drzewoski J (2004). DNA damage and repair in type 2 diabetes mellitus. Mutation research.

[53] Jeggo PA, Lobrich M (2007). DNA double-strand breaks: their cellular and clinical impact?. Oncogene.

[54] Ornoy A (2005). Growth and neurodevelopmental outcome of children born to mothers with pregestational and gestational diabetes. Pediatric endocrinology reviews.

[55] Moreli JB, Santos JH, Rocha CR, Damasceno DC, Morceli G, Rudge MV, Bevilacqua E, Calderon IM (2014). DNA damage and its cellular response in mother and fetus exposed to hyperglycemic environment. BioMed research international.

[56] Savitsky K, Bar-Shira A, Gilad S, Rotman G, Ziv Y, Vanagaite L, Tagle DA, Smith S, Uziel T, Sfez S, Ashkenazi M, Pecker I, Frydman M (1995). A single ataxia telangiectasia gene with a product similar to PI-3 kinase. Science.

[57] Qiu H, Lee S, Shang Y, Wang WY, Au KF, Kamiya S, Barmada SJ, Finkbeiner S, Lui H, Carlton CE, Tang AA, Oldham MC, Wang H (2014). ALS-associated mutation FUS-R521C causes DNA damage and RNA splicing defects. J Clin Invest.

[58] Wang WY, Pan L, Su SC, Quinn EJ, Sasaki M, Jimenez JC, Mackenzie IR, Huang EJ, Tsai LH (2013). Interaction of FUS and HDAC1 regulates DNA damage response and repair in neurons. Nat Neurosci.

[59] Suberbielle E, Djukic B, Evans M, Kim DH, Taneja P, Wang X, Finucane M, Knox J, Ho K, Devidze N, Masliah E, Mucke L (2015). DNA repair factor BRCA1 depletion occurs in Alzheimer brains and impairs cognitive function in mice. Nature communications.

[60] Pulvers JN, Huttner WB (2009). Brca1 is required for embryonic development of the mouse cerebral cortex to normal size by preventing apoptosis of early neural progenitors. Development.

[61] Kovacs GG, Adle-Biassette H, Milenkovic I, Cipriani S, van Scheppingen J, Aronica E (2014). Linking pathways in the developing and aging brain with neurodegeneration. Neuroscience.

[62] Gokozan HN, Baig F, Corcoran S, Catacutan FP, Gygli PE, Takakura AC, Moreira TS, Czeisler C, Otero JJ (2015). Area postrema undergoes dynamic postnatal changes in mice and humans. The Journal of comparative neurology.

[63] Chang JC, Leung M, Gokozan HN, Gygli PE, Catacutan FP, Czeisler C, Otero JJ (2015). Mitotic events in cerebellar granule progenitor cells that expand cerebellar surface area are critical for normal cerebellar cortical lamination in mice. J Neuropathol Exp Neurol.

[64] Jagers P (1975). Branching processes with biological applications.

[65] Till JE, McCulloch EA, Siminovitch L (1964). A Stochastic Model of Stem Cell Proliferation, Based on the Growth of Spleen Colony-Forming Cells. Proc Natl Acad Sci U S A.

[66] Lambert A (2005). The Branching Process with Logistic Growth. The Annals of Applied Probability.

[67] Bellman R, Harris T (1952). On Age-Dependent Binary Branching Processes. Annals of Mathematics.

[68] Noctor SC, Flint AC, Weissman TA, Dammerman RS, Kriegstein AR (2001). Neurons derived from radial glial cells establish radial units in neocortex. Nature.

[69] Lien WH, Klezovitch O, Fernandez TE, Delrow J, Vasioukhin V (2006). alphaE-catenin controls cerebral cortical size by regulating the hedgehog signaling pathway. Science.

[70] Breunig JJ, Silbereis J, Vaccarino FM, Sestan N, Rakic P (2007). Notch regulates cell fate and dendrite morphology of newborn neurons in the postnatal dentate gyrus. Proc Natl Acad Sci U S A.

[71] Nyfeler Y, Kirch RD, Mantei N, Leone DP, Radtke F, Suter U, Taylor V (2005). Jagged1 signals in the postnatal subventricular zone are required for neural stem cell self-renewal. EMBO J.

[72] Pearl R, Reed LJ (1920). On the Rate of Growth of the Population of the United States since 1790 and Its Mathematical Representation. Proc Natl Acad Sci U S A.

[73] Gilles FH, Leviton A, Dooling EC (2013). The Developing Human Brain: Growth and Epidemiologic Neuropathology.

[74] Olive PL, Banath JP (2006). The comet assay: a method to measure DNA damage in individual cells. Nat Protoc.

[75] Gyori BM, Venkatachalam G, Thiagarajan PS, Hsu D, Clement MV (2014). OpenComet: An automated tool for comet assay image analysis. Redox biology.

[76] Rousseau L, Etienne O, Roque T, Desmaze C, Haton C, Mouthon MA, Bernardino-Sgherri J, Essers J, Kanaar R, Boussin FD (2012). In vivo importance of homologous recombination DNA repair for mouse neural stem and progenitor cells. PLoS One.

[77] Paull TT, Rogakou EP, Yamazaki V, Kirchgessner CU, Gellert M, Bonner WM (2000). A critical role for histone H2AX in recruitment of repair factors to nuclear foci after DNA damage. Curr Biol.

[78] Sunyer B, Patil S, Höger H, Lubec G (2007). Barnes maze, a useful task to assess spatial reference memory in the mice.

[79] Deacon RM (2013). Measuring motor coordination in mice. Journal of visualized experiments.

[80] Vermeirsch H, Meert TF (2004). Morphine-induced analgesia in the hot-plate test: comparison between NMRI(nu/nu) and NMRI mice. Basic Clin Pharmacol Toxicol.

[81] Fotaki V, Martinez De Lagran M, Estivill X, Arbones M, Dierssen M (2004). Haploinsufficiency of Dyrk1A in mice leads to specific alterations in the development and regulation of motor activity. Behav Neurosci.

[82] Yang M, Crawley JN, Crawley Jacqueline N (2009). Simple behavioral assessment of mouse olfaction. Current protocols in neuroscience.

[83] Bailey KR, Crawley JN, Buccafusco JJ (2009). Anxiety-Related Behaviors in Mice. Methods of Behavior Analysis in Neuroscience.

[84] Walf AA, Frye CA (2007). The use of the elevated plus maze as an assay of anxiety-related behavior in rodents. Nat Protoc.

[85] Budzynska B, Boguszewska-Czubara A, Kruk-Slomka M, Skalicka-Wozniak K, Michalak A, Musik I, Biala G, Glowniak K (2013). Effects of imperatorin on nicotine-induced anxiety- and memory-related responses and oxidative stress in mice. Physiol Behav.

[86] Deacon RM (2006). Digging and marble burying in mice: simple methods for in vivo identification of biological impacts. Nat Protoc.

[87] Can A, Dao DT, Arad M, Terrillion CE, Piantadosi SC, Gould TD (2012). The mouse forced swim test. Journal of visualized experiments.

[88] Can A, Dao DT, Terrillion CE, Piantadosi SC, Bhat S, Gould TD (2012). The tail suspension test. Journal of visualized experiments.

