# Supplementary material for: Cyclin A2 promotes DNA repair in the brain during both development and aging
Source: Aging (Albany NY). 2016 Jul 13;8(7):1540–64. doi: 10.18632/aging.100990 (PMC4993346; doi:10.18632/aging.100990)
Supplement: Supplementary file 1 [file aging-08-1540-s001.pdf]

SUPPLEMENTAL DATA

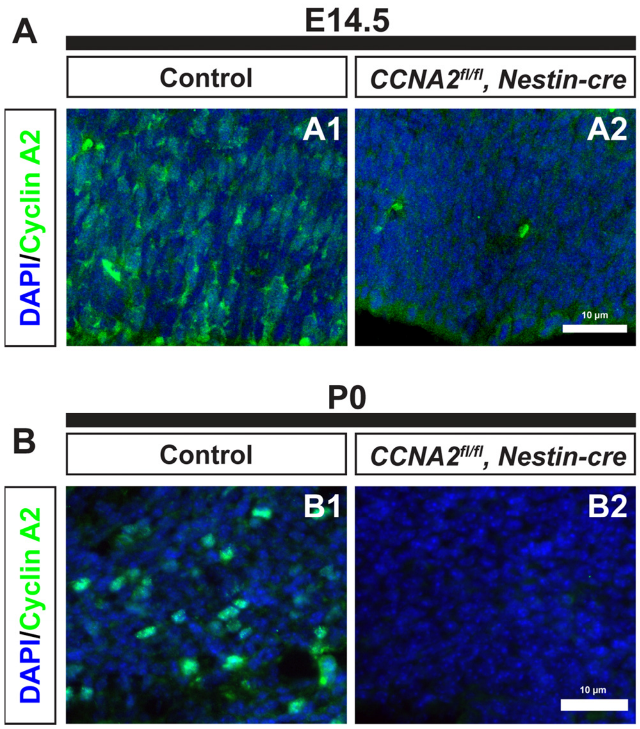

Figure S1. Ablation of *CCNA2* by Cre. *CCNA2[fl/fl]* mice were bred to *Nestin-cre* mice resulting in almost complete knockout of *CCNA2* expression by E14.5 (**A**), which continued into postnatal ages (**B**).

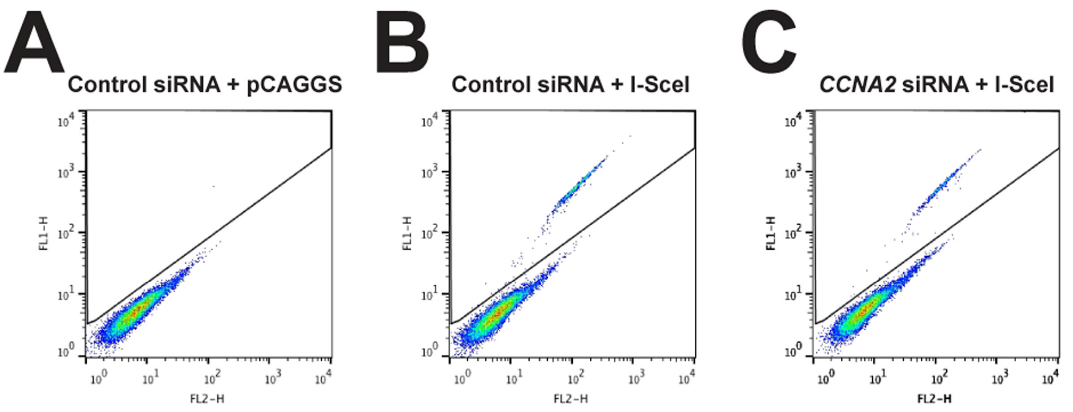

Figure S2. Gating strategy for HR and NHEJ assays. (**A**) Cells with no GFP expression were used for gating to identify GFP+ cells in control (**B**) or *CCNA2*-silenced cells (**C**). The *x*-axis represents red fluorescence, and the *y*-axis represents green fluorescence. Plots are from a representative DRGFP HR assay experiment.

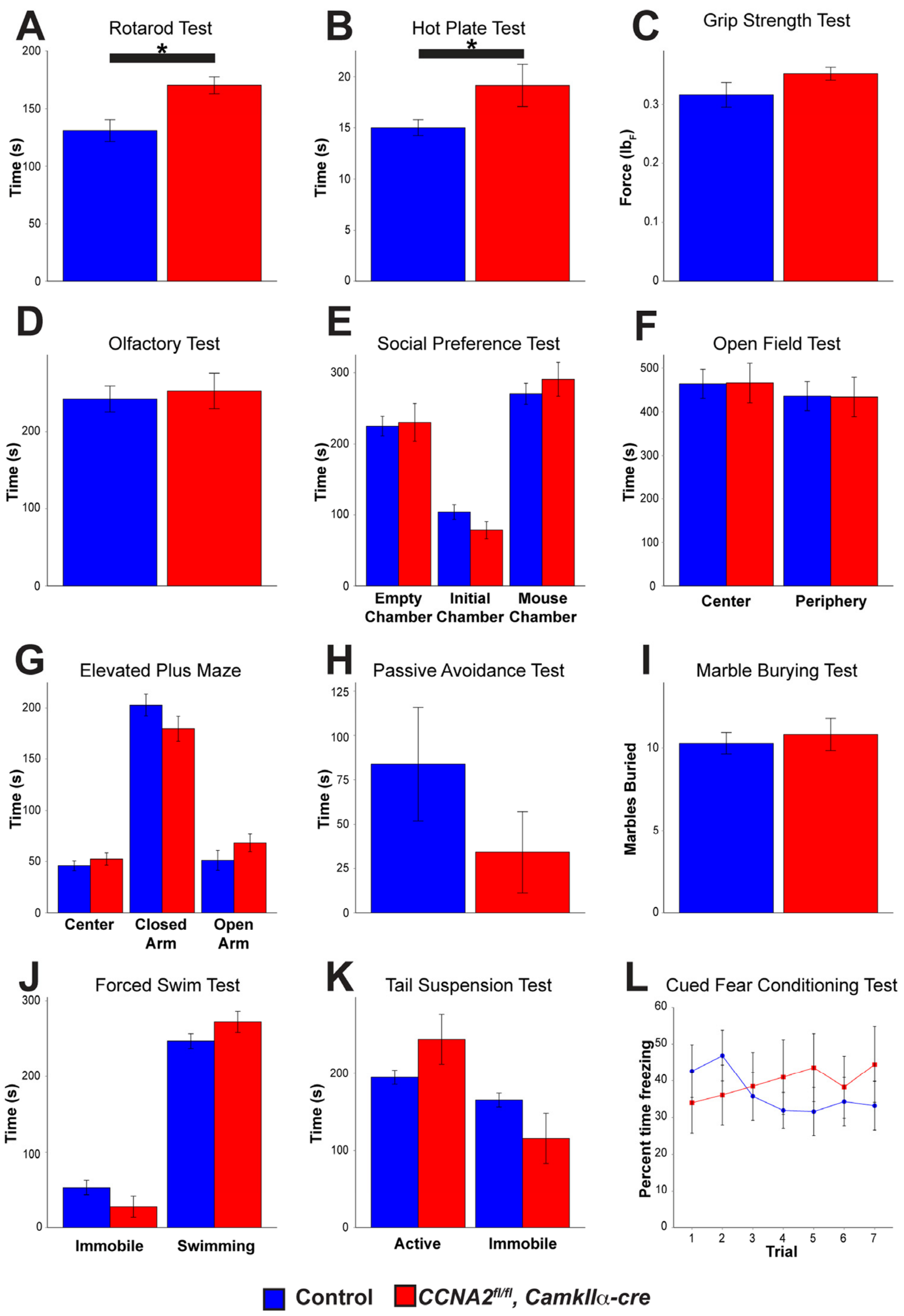

Figure S3. Behavioral analysis of *CCNA2$^{fl/fl}$*, *CamkIIα-cre* mice. Mice were subjected to a battery of behavioral tests. Significant, although minor, differences were observed in Rotarod performance (**A**) and hot plate pain perception tests (**B**). Other tests showed no significant differences, including grip strength (**C**), olfactory test (**D**), sociability tests (**E**), open field test (**F**), elevated plus maze (**G**), passive avoidance test (**H**), marble burying (I), Porsolt forced swim test (**J**), tail suspension test (**K**), and cued fear conditioning (**L**). Unpaired *t*-test, * = $p < 0.05$. n = 12 control mice and 7 *CCNA2$^{fl/fl}$*, *CamkIIα-cre* mice. Error bars represent s.e.m.

Table S1. Upper and lower limits of apoptosis levels in the cerebellar EGL and forebrain VZ/SVZ.

	% Apoptotic Volume		
**EGL**	**Control**	***CCNA2$^{fl/fl}$*, *Nestin-cre***	**Fold Change**
E14.5	0.11-0.46%	1.43-5.74%	12.6
E17.5	0.24-0.96%	0.96-3.9%	4.0
**Forebrain VZ/SVZ**			
E14.5	0.015-0.061%	0.40-1.60%	26.3
E17.5	0.0052-0.021	0.015-0.061	2.9

Values represent the percent volume of each structure that is occupied by apoptotic cells in the forebrain and cerebellar stem cell niches. Apoptotic volumes for the forebrain VZ/SVZ were calculated using values presented in Fig. 1. Volumes for the cerebellar EGL were calculated from previously published values from Otero, et al. [1]. We used an expected cellular volume of 200-800 μm^3 as described for spinal progenitors [2], which is expected to be similar to other progenitor cells. Levels are presented as volumes of the structure represented by apoptotic cells for consistency with our model.

Table S2. Variables and descriptions.

Variable	Description
$G1_{RG}^{(k)}$	Volume of radial glia neural progenitors of age k in G1 phase (of any cell fate)
$S_{RG}^{(k)} S_{RG}^{(k)'}, S_{RG}^{(k)''}$	Volume of radial glia of age k in S phase that are currently proliferative/self-renewing/differentiating
$G2_{RG}^{(k)}, G2_{RG}^{(k)'}, G2_{RG}^{(k)''}$	Volume of radial glia of age k in G2 phase that are currently proliferative/self-renewing/differentiating
$M_{RG}^{(k)}, M_{RG}^{(k)'}, M_{RG}^{(k)''}$	Volume of radial glia of age k in M phase that are currently proliferative/self-renewing/differentiating
$G1_{IP}$	Volume of intermediate progenitors in G1 phase
S_{IP}	Volume of intermediate progenitors in S phase
$G2_{IP}$	Volume of intermediate progenitors in G2 phase
M_{IP}	Volume of intermediate progenitors in M phase
A_{VZ}	Volume of apoptotic cells in the VZ
A_{SVZ}	Volume of apoptotic cells in the SVZ
A_{CP}	Volume of apoptotic cells present in the cortical plate
$G0$	Volume of post-mitotic neurons (assumed to be in the progress of migrating to the cortical plate).
CP	Volume of cortical plate

Table S2. Variables and descriptions.

Equation	Description
$\frac{dG_{1RG}^{(k)}}{dt} = 2J_M^{(k-1)} + J_M^{(k-1)'} - J_{G1}^{(k)} - J_{G1}^{(k)'} - J_{G1}^{(k)''} - J_{AG1}^{(k)}$	The rate of change in the volume occupied by G1 phase radial glia of age k.
$\frac{dS_{RG}^{(k)}}{dt} = J_{G1}^{(k)} - J_S^{(k)} - J_{AS}^{(k)}$	The rate of change in the volume occupied by S phase radial glia of age k committed to symmetric self-renewal.
$\frac{dS_{RG}^{(k)'}}{dt} = J_{G1}^{(k)'} - J_S^{(k)'} - J_{AS}^{(k)'}$	The rate of change in the volume occupied by S phase radial glia of age k committed to asymmetric self-renewal.
$\frac{dS_{RG}^{(k)''}}{dt} = J_{G1}^{(k)''} - J_S^{(k)''} - J_{AS}^{(k)''}$	The rate of change in the volume occupied by S phase radial glia of age k committed to non-self-renewal.
$\frac{dG_{2RG}^{(k)}}{dt} = J_S^{(k)} - J_{G2}^{(k)} - J_{AG2}^{(k)}$	The rate of change in the volume occupied by G2 phase radial glia of age k committed to symmetric self renewal.
$\frac{dG_{2RG}^{(k)'}}{dt} = J_S^{(k)'} - J_{G2}^{(k)'} - J_{AG2}^{(k)'}$	The rate of change in the volume occupied by G2 phase radial glia of age k committed to asymmetric self-renewal.
$\frac{dG_{2RG}^{(k)''}}{dt} = J_S^{(k)''} - J_{G2}^{(k)''} - J_{AG2}^{(k)''}$	The rate of change in the volume occupied by G2 phase radial glia of age k committed to non-self-renewal.
$\frac{dM_{RG}^{(k)}}{dt} = J_{G2}^{(k)} - J_M^{(k)} - J_{AMRG}^{(k)}$	The rate of change in the volume occupied by M phase radial glia of age k committed to symmetric self renewal.
$\frac{dM_{RG}^{(k)'}}{dt} = J_{G2}^{(k)'} - J_M^{(k)'} - J_{AMRG}^{(k)'}$	The rate of change in the volume occupied by M phase radial glia of age k committed to asymmetric self-renewal.
$\frac{dM_{RG}^{(k)''}}{dt} = J_{G2}^{(k)''} - J_M^{(k)''} - J_{AMRG}^{(k)''}$	The rate of change in the volume occupied by M phase radial glia of age k committed to non-self-renewal.
$\frac{dG1_{IP}}{dt} = J_M^{(K)''} - J_{IPG1} - J_{AIPG1}$	The rate of change in the volume occupied by G1 phase intermediate progenitors.
$\frac{dS_{IP}}{dt} = J_{IPG1} - J_{IPS} - J_{AIPS}$	The rate of change in the volume occupied by S phase intermediate progenitors.
$\frac{dG2_{IP}}{dt} = J_{IPS} - J_{IPG2} - J_{AIPG2}$	The rate of change in the volume occupied by G2 phase intermediate progenitors.

$\frac{dM_{IP}}{dt} = J_{IPG2} - J_{IPM} - J_{AIPM}$	The rate of change in the volume occupied by M phase intermediate progenitors.
$\frac{dA_{CP}}{dt} = J_{ACP} - \frac{A_{CP}}{t_{clear}}$	The rate of change in the volume occupied by apoptotic cells in the cortical plate.
$\frac{dA_{VZ}}{dt} = \sum_k \left[J_{AG1}^{(k)} + J_{AS}^{(k)} + J_{AS}^{(k)'} + J_{AG2}^{(k)} + J_{AG2}^{(k)'} + J_{AG2}^{(k)''} + J_{AMRG}^{(k)} + J_{AMRG}^{(k)'} + J_{AMRG}^{(k)''}\right] - \frac{A_{VZ}}{t_{clear}}$	The rate of change in the volume occupied by apoptotic cells in the ventricular zone.
$\frac{dA_{SVZ}}{dt} = J_{AIPG1} + J_{AIPS} + J_{AIPG2} + J_{AIPM} - \frac{A_{SVZ}}{t_{clear}}$	The rate of change in the volume occupied by apoptotic cells in the subventricular zone.
$\frac{dG0}{dt} = \sum_k \left[J_M^{(k)'} + J_M^{(k)}{''}\right] - J_{migrate} + 2J_{IPM} - J_{AG0}$	The rate of change in the volume of migratory post-mitotic cells.
$\frac{dCP}{dt} = J_{migrate} - J_{ACP}$	The rate of change in the volume of the cortical plate.

Table S4. Flows between the states.

Rates	Description/Comments	Value
$J_M^{(k)}, J_M^{(k)'}, J_M^{(k)''}$	Proliferative/self-renewing/differentiating RG cells of age k exiting M to produce cells of age $k+1$.	$\frac{M_{RG}^{(k)}}{t_M}; \frac{M_{RG}^{(k)'}}{t_M}; \frac{M_{RG}^{(k)''}}{t_M}$
$J_{G1}^{(k)}, J_{G1}^{(k)'}, J_{G1}^{(k)''}$	RG cells of age k exiting G1 with cell fates determined to be proliferative/self-renewing/differentiating	$k < K$: $p\frac{G1_{RG}^{(k)}}{t_{G1P}}; (1-p)\frac{G1_{RG}^{(k)}}{t_{G1R}}; 0$ $k = K$: $0,0,\frac{G1_{RG}^{(K)}}{t_{G1D}}$
$J_S^{(k)}, J_S^{(k)'}, J_S^{(k)''}$	Proliferative/self-renewing/differentiating RG cells of age k exiting S	$\frac{S_{RG}^{(k)}}{t_S}, \frac{S_{RG}^{(k)'}}{t_S}, \frac{S_{RG}^{(k)''}}{t_S}$
$J_{\mathbf{G2}}^{(k)}, J_{G2}^{(k)'}, J_{G2}^{(k)''}$	Proliferative/self-renewing/differentiating RG cells of age k leaving G2	$\frac{G2_{RG}^{(k)}}{t_{G2}}, \frac{G2_{RG}^{(k)'}}{t_{G2}}, \frac{G2_{RG}^{(k)''}}{t_{G2}}$
J_{IPG1}	IP cells leaving G1	$\frac{G1_{IP}}{t_{G1IP}}$
J_{IPS}	IP cells leaving S	$\frac{S_{IP}}{t_S}$
J_{IPG2}	IP cells leaving G2	$\frac{1}{t_{G2}} G2_{IP}$
J_{IPM}	IP cells leaving M	$\frac{1}{t_M} M_{IP}$

Table S5. Parameter values for model.

Parameter	Control	*CCNA2*$^{fl/fl}$, *Nestin-cre* (if different)
t_{G1RG}	$3.2\ hr$	--
t_{G1IP}	$12.1\ hr$	--
t_{G1D}	$18.2\ hr$	--
t_{G2}	$1.6\ hr$	$2.0\ hr$
t_M	$0.4\ hr$	--
t_S	$4.0\ hr$	$5.0\ hr$
V_{max}	$1.2 \times 10^9 \mu m^3$	--
K	5	6
t_{clear}	2.33	2.33

Timing parameter values are taken from Takahashi et al. [3]. The carrying capacity of the VZ was chosen to yield correct qualitative behavior for the development of the VZ/SVZ layers. Values for *CCNA2*$^{fl/fl}$, *Nestin-cre* animals are presented if different from the control and were calculated from our unbiased stereology data using our mathematical model.

Supplemental Glossary

Logistic growth: Population growth whereby the growth rate of a population is relative to its size. Furthermore, the growth rate decreases as the population saturates its niche and reaches its carrying capacity.

Carrying capacity : The maximum population of a given niche, whether for stem cells or for a population of animals, for example. Carrying capacity is determined by a number of factors, including space and other available resources.

Ordinary differential equations: Equations describing the rate of change of particular quantities relative to changes in other quantities (e.g. population vs. size)

Parameterize: We use experimentally provided values provided by our and others' experiments in order to set the constants embedded in our ordinary differential equations.

Population Dynamics: The dynamics in terms of population size of the individual niches that form the developing VZ/SVZ.

Unbiased stereology: A methodology utilized to determine without bias measurements from three dimensional structures from a serial collection of randomly sampled but serially aligned 2-dimensional structures. In our iteration, we use an automated stage driven by Stereoinvestigator.

Supplemental References

1. Otero JJ, Kalaszczynska I, Michowski W, Wong M, Gygli PE, Gokozan HN, Griveau A, Odajima J, Czeisler C, Catacutan FP, Murnen A, Schuller U, Sicinski P, et al. Cerebellar cortical lamination and foliation require cyclin A2. Dev Biol. 2014; 385:328-39.

2. McMahon SS, Dockery P and McDermott KW. Estimation of nuclear volume as an indicator of maturation of glial precursor cells in the developing rat spinal cord: a stereological approach. J Anat. 2003; 203:339-44.

3. Takahashi T, Nowakowski RS and Caviness VS, Jr. The leaving or Q fraction of the murine cerebral proliferative epithelium: a general model of neocortical neuronogenesis. J Neurosci. 1996; 16:6183-96.
